# Melatonin improves endometrial receptivity and embryo implantation via MT2/PI3K/LIF signaling pathway in sows

**DOI:** 10.1186/s40104-024-01137-x

**Published:** 2025-01-04

**Authors:** Xue Qin, Menghao Yang, Yang Yu, Xiaolin Wang, Yi Zheng, Rui Cai, Weijun Pang

**Affiliations:** Key Laboratory of Northwest China’s Pig Breading and Reproduction, Ministry of Agriculture and Rural Affairs of the People’s Republic of China, College of Animal Science and Technology, Northwest A&F University, Yangling, 712100 Shaanxi China

**Keywords:** Backfat thickness, Embryo implantation, Endometrial receptivity, Melatonin, MT2/PI3K/LIF, Sow

## Abstract

**Background:**

Increased backfat thickness of sows in early gestation is negative to reproductive performance. Endometrial receptivity is an important determinant of reproductive success, but it is unclear whether the effect of sow backfat thickness on litter size is associated with endometrial receptivity and whether melatonin treatment may have benefits. The present study seeks to answer these questions through in vitro and in vivo investigations.

**Results:**

Excessive lipid deposition and lower melatonin levels in the uterus are detrimental to endometrial receptivity and embryo implantation in high backfat thickness sows. In cells treated with melatonin, the MT2/PI3K/LIF axis played a role in reducing lipid accumulation in porcine endometrial epithelium cells and improved endometrial receptivity. Furthermore, we found a reduction of lipids in the uterus after eight weeks of intraperitoneal administration of melatonin to HFD mice. Notably, melatonin treatment caused a significant reduction in the deposition of endometrial collagen, an increase in the number of glands, and repair of the pinopode structure, ultimately improving endometrial receptivity, promoting embryo implantation, and increasing the number of litter size of mice.

**Conclusions:**

Collectively, the finding reveals the harmful effects of high backfat thickness sows on embryo implantation and highlight the role of melatonin and the MT2/PI3K/LIF axis in improving endometrial receptivity by enhancing metabolism and reducing the levels of uterine lipids in obese animals.

**Supplementary Information:**

The online version contains supplementary material available at 10.1186/s40104-024-01137-x.

## Background

Backfat thickness reflects the nutritional status and physical reserves of sows at different stages, and there is a strong correlation between it and reproductive performance. Sows with excessive backfat may develop large fat deposits in the abdomen and uterus, which may reduce uterine function. Previous studies have shown that mammal with overweight or obesity can conceive naturally, but they have reduced fecundability [1–3]. It is possible that maternal obesity may impair several reproductive processes, including follicular development, endometrial receptivity and embryo implantation [4–8]. A considerable number of mammals who are obese continue to face implantation failure even after high-quality embryo transferred, this implicates abnormalities in the endometrium and its function in impaired reproductive performance in such individuals [6, 9–11]. That is, the embryos may have been transferred to an unacceptable endometrium, as displacement of the window of implantation is known to increase in a BMI-dependent manner [12]. Despite the achievements in improving gamete quality and insemination techniques, there is still much work to be done in terms of better understanding endometrial functions [13]. Thus, the question of whether endometrial receptivity plays a larger role in low fecundity in sow with high backfat thickness still remains to be answered.

Embryo implantation is the process by which the embryo migrates, localizes and attaches itself to the endometrium and gradually establishes a physiological connection with the maternal uterus, which is essential for the gestation of the sow [14–16]. Embryo implantation depends on a receptive endometrium, activated blastocyst, and the synchronization between them. In fact, endometrium receptivity as a determinant factor and is particularly significant for studying maternal–fetal crosstalk [17–19]. Investigating the evolution of the signaling axis related to obesity [20] and the regulation of endometrial receptivity might contribute to reveal on the relationship between the two in a more precise way. For example, the AMPK [21], PI3K [22], MAPK [23, 24], JAK/STAT [25] and Wnt/β-catenin [26, 27] signaling pathways are involved in appetite regulation, metabolic homeostasis and lipid deposition. In addition, the ACVR2A-SMAD1/SMAD5 [28], PI3K/AKT [29], Wnt/β-catenin [30], ERK1/2-mTOR [31] and NF-κB/ZEB1/E-Cadherin [32] signaling pathways have been explored for their regulatory function in endometrial receptivity. Thus, targeting these pathways and their molecules may be crucial for the exploration of the impact of lipids on uterine function.

Endometrial receptivity is susceptible to various factors [19, 33–36] among which energy metabolism is a crucial factor related to proper endometrial function. The evidence accumulated so far highlights the primary role of metabolism in endometrial receptivity and implies that reduced metabolism caused by obesity may reduce endometrial receptivity [37, 38]. Thus, treatment with exogenous hormones could help balance metabolism and improve fertility in animals with obesity. In this light, melatonin regulates fat metabolism, lipolysis, and blood glucose stabilization, making it an effective medication for the treatment of obesity. Further, melatonin supplements are thought to be beneficial for alleviating metabolic dysregulation [39, 40]. Furthermore, as uterine activities are cyclical, melatonin is essential to regulate development of endometrial cells, regulation of metabolic status and free radical scavenging and maintenance of vascular dynamics [41, 42]. Hence, the success of mammalian reproduction may be related to melatonin levels in the uterus.

Therefore, it is necessary to investigate whether low litter size in high backfat sows is closely related to endometrial receptivity. Meanwhile, exploring a signaling axis that melatonin regulates the function of the endometrium may help improve endometrial receptivity and embryo implantation in obese sows. These findings provide new insights into potential signaling pathways for maternal-conceptus interface during early pregnancy in sow.

## Materials and methods

### Animal and experimental design

Third-parity crossbred (Large White × Landrace) sows generally having an average weight ranging from 190 to 240 kg were raised at Xinjiang Xukang Breeding Co., Ltd. (Gansu, China) slaughtered at Pingao Food Co., Ltd. (Gansu, China). The age of sow are typically around 2 to 2.5 years. Sows with similar parity and genetic background were divided into the control group (normal backfat thickness, *n* = 6) and obese group (high backfat thickness, *n* = 6) according to visual Body Condition Scoring (BCS) [43]. All the sows were artificially inseminated twice and were slaughtered 13 d after insemination. The uterus was flushed with saline and the morphology of the blastocysts and the concentration of progesterone in the serum were observed to confirm that the mated sows were pregnant. Measurement of backfat thickness and uterine weight. Further, the uterus, ovaries and uterine cavity fluid were collected. Blood samples were collected from anterior vena cava and the serum component was stored at −80 °C.

For the mouse models, female mice (C57BL/6 J, 8 weeks old, 19 g) were purchased from Chengdu Yaokang Biotechnology Co., Ltd. (Chengdu, China) and provided with a control diet (*n* = 40, Cat# GB14924.3: 12.7% fat energy) or a high-fat diet (*n* = 40, Cat# H10060: 60% fat energy) ad libitum last for 3 months beginning at 8 weeks of age, which made the body weight of HFD mice reach 30–35 g. The mice were housed in the Animal Breeding Center of Northwest A&F University and were free to drink and feed.

For the melatonin treatment experiments, melatonin dissolved in saline to a final concentration and intraperitoneally injected at a dose of 20 mg/kg/d for 2 months. An equal volume of saline was used as the control. The experiment was divided into four groups, a control + NaCl group (mice with injection of saline, *n* = 20), a control + Mel group (mice with injection of melatonin, *n* = 20), an HFD + NaCl group (HFD mice with injection of saline, *n* = 20), and an HFD + Mel group (HFD mice with injection of melatonin, *n* = 20). Male mice used for breeding the females were fed the control diet. Observed with vaginal plugs to localize pregnancy for 0.5 d, 6 female mice per group were anesthetized at 5.5 d, and 1% Evan’s blue dye (CAS No.: 2610-05-1 C8679, Sigma) dissolved in saline solution was injected tail vein. The mice were sacrificed 2 h after injection, and uterus and serum samples were obtained. The embryonic attachment sites in the uteri were noted, and the number of embryos slaughtered at 16.5 d of gestation was counted. Uterus, ovaries and serum samples were collected. And we counted the number of pups born on the third day of the female mice.

### Cell culture

The porcine endometrial epithelial cells (PEECs) were purchased from Weijia Technology Co., Ltd. (Guangzhou, China). PEECs were cultured in culture medium (Primed-icell-001, Shaanxi, China) at 37 °C and 5% CO_2_. Cells were cultured in 100 mm Petri dishes. When the cells grew to 80%−90%, they were passed into the 6/12/24/96-well plate for cell treatment. Then, dilute palmitic acid (PA) without fetal bovine serum medium to which PA was added at a concentration of 40 μmol/L and cultured for 48 h (PA group). A concentration of 20 μmol/L of melatonin was added after 24 h of treatment with PA (PA + Mel group). In the last group, that is, the Mel group, only melatonin was added at a concentration of 20 μmol/L for 48 h. The medium was changed every 48 h. The melatonin solution (TargeMol, Shanghai, China) was configured using dimethyl sulfoxide (DMSO) to 10 mmol/L (C6164, Solarbio, Beijing, China). The final concentrations were determined according to the requirements of the experiments. The inhibition studies used the nonselective melatonin receptor antagonist luzindole (CAS No.: HY-101254, MCE), the selective MT2 blocker 4P-PDOT (CAS No.: HY-100609, MCE), and the PI3K inhibitor wortmannin (CAS No.: HY-10197, MCE). The cells were first pre-treated with inhibitor-containing medium for 1 h, then replace it with medium containing the inhibitors, PA or melatonin, as per the experimental setup.

### CCK-8 assay

Cell viability was detected with the CCK-8 assay (Cat No.: C6030, New Cell & Molecular Biotech Co., Ltd., Suzhou, China). PEECs were treated with palmitic acid (0, 20, 40, 60, 80, and 100 μmol/mL) [44–46] (P0500, Sigma) for 24 h, and pre-treated with melatonin (0, 5, 10, 15, 20, and 25 μmol/mL) [47, 48] (M5250, Sigma) at 37 °C for 24 h in 96-well plate (*n* = 4 biological replicates). Then, CCK-8 reagent was added and the absorbance at 450 nm was then detected using an enzyme meter (Multiskan FC, Thermo Scientific).

### EdU assay

Proliferation of PEECs was quantified using an EdU kit in 48-well plate (*n* = 3 biological replicates) (RIBOBIO RN: R11056.6). EdU‐positive cells number were observed by using fluorescence microscope. Three regions per group were randomly selected for statistical analysis.

### Oil Red O staining

Consistent with our team’s experimental procedure [49], in short, PEECs were washed with PBS (Gibco) and fixed in 4% paraformaldehyde solution (Solarbio, Beijing, China) in 6-well plate (*n* = 5 biological replicates). Subsequently, the wells were rinsed with PBS and stained with Oil Red O (Sigma) for 30 min. After rinsing with PBS, the size and area of the lipid droplets were observed using microscopically (Olympus, Tokyo, Japan). PEECs were treated with isopropanol, then pipette tips were blown repeatedly to dissolve the intracellular Oil Red O reagent, and absorbance was measured at 490 nm to further quantify the lipid droplet content.

### ELISA assay

Detection of melatonin levels in serum (*n* = 5) and uterine fluid (*n* = 6) of sows by using porcine and mice MLT ELISA kits, respectively (JM-10394P1, JM-02510M1; Jiangsu Jingmei Biological Technology Co., Ltd., China). The range of standard curve of Melatonin (1.0–80 pg/mL). Intra-/inter-assay coefficients of variation for the ELISA were all less than 15% (Melatonin).

### Wound healing assay

Cells were cultured in 6-well plates to 90%−100% confluence, and uniform scratches were made on the cell monolayers using a sterile 200 μL pipette tip, which was gently washed with PBS to remove floating cells. Microscopic photographs were taken at 0, 12, 24, and 36 h after trauma formation to record the width of the scratches (*n* = 4 per group). Image J software was used to calculate the scratch size, and the final use was (initial cell scratch distance − scratch distance at observation)/interval time.

### RNA extraction and reverse transcription

Consistent with our team’s operational steps [50], briefly, the total RNA was extracted with TRIzol reagent (Invitrogen). RNA purity was confirmed by OD_260_/OD_280_ ratios and the concentration of RNA was measured using a spectrophotometer (Thermo Fisher Scientific, Waltham, MA, USA), then reverse transcribed for qPCR (R323-01, Vazyma).

### Quantitative real-time PCR

Primers were designed using Primer5 software and verified for specificity via melt curve analysis. And our primers in qRT-PCR were designed to span exon boundaries to avoid amplification of contaminating genomic DNA. qRT-PCR was performed using 2 × SYBR Green qPCR Master Mix (Q311-02, Vazyma) with a StepOnePlus Real-Time PCR System, with the following thermal profile: 95 °C for 10 min, followed by 40 cycles of 95 °C for 15 s and 60 °C for 1 min. The expression of target genes was analyzed using two internal references, β-actin and GAPDH, respectively. Relative mRNA levels were calculated using the 2^-ΔΔCt^ method. PCR reaction efficiencies between 90% and 110%, absolute slopes of standard curves between 3.0 and 3.6. The negative controls in qRT-PCR were No Template Control (NTC). Table S1 demonstrates the primer sequences used in this experiment.

### Western blotting

PEECs or uteri were lysed with RIPA buffer containing protease (1:100) and phosphatase inhibitor (1:100) (M7528, Lihe Biology). Electrophoresis conditions of 80 V (20 min) and 120 V (1 h) were applied, followed by wet-turn transfer to a PVDF membrane (IPVH0010, Chennuo Biology). The primary antibody was incubated at 4 °C for 10–12 h, and the secondary antibody was incubated at room temperature for 1 h. The gel concentration used was 10%, and the sample size was about 25 μg. Bands were analyzed for gray values using Image J software and relative protein expression levels were normalized to β-actin expression levels (*n* = 3 biological replicates). Antibody information is in Table S2.

### Si-RNA knockdown experiments

For the gene silencing assays, transfection was performed using Lipofectamine RNAi (Invitrogen) in 6-well plates (*n* = 4). For the knockdown assays, siMT1 (Tong Yong, Anhui, China), siMT2 (Tong Yong, Anhui, China), siLIF (Tong Yong, Anhui, China), and control siRNA (Tong Yong, Anhui, China) were used. For each experiment, Lipofectamine RNA iMax was diluted with a reduced serum medium (Opti‐MEM; Invitrogen) and then mixed with siRNA. The cells were returned to the incubator and replaced with an FBS-containing medium within 12 h after transfection to improve cell survival. qRT-PCR and Western blot were used to detect interference efficiency.

### Immunofluorescence analysis

Protein expression of FASN, FABP4, Integrin β3, VEGFA, LIF, and PR in PEECs was detected using immunocytochemistry. Briefly, the PEECs were fixed, washed and permeabilized. Incubate overnight at 4 °C with primary antibodies such as FASN, FABP4, Integrin β3, VEGFA, LIF and PR. Incubate the secondary antibody for 2 h. Nuclei were stained with DAPI (Solarbio, Beijing, China) for 15 min and visualized with microscope. Negative control for immunofluorescence was the no primary antibody control. Fluorescence intensity analysis was performed using Image J, *n* = 5 per group. Antibody information is in Table S2.

### RNA-seq and data analyses

Total RNA from the uterine samples of obese and control sows (four samples per group) were extracted using TRIzol (Invitrogen, Carlsbad, CA, USA) and then treated with DNaseI to eliminate genomic DNA. The integrity of the extracted RNA was assessed using Agilent Bioanalyzer. Next, RNA libraries were prepared using Illumina’s TruSeq Kit and were quantified and sequenced on an Illumina HIseq2000 instrument for paired-end reads. The RNA-seq data underwent initial quality control with FastQC, with Trimmomatic used for trimming adapters and low-quality bases. Differential expression analysis was conducted using DESeq2, and GO and KEGG pathway analyses were used to explore the critical biological processes that were enriched for the differentially expressed genes identified. For KEGG pathways enrichment, we based on unadjusted *P*-values < 0.05; for volcano plot, we used adjusted *P*-values to control the false discovery rate (FDR). Genes with a −log_10_ (*P* -value) greater than 1 and a log_2_ fold change (log_2_FC) greater than 1 or less than −1 were considered significantly differentially expressed. All the data analyses were performed using the R software.

### Infrared thermography

Mice temperature was measured at room temperature, using an infrared camera (FLIR Systems). Several infrared pictures were taken of every mouse during the light phase, while it was allowed to move freely on a cage lid. Body surface temperatures were measured using a thermal imager (FLIR systems, Wilsonville, OR, USA) and analyzed using FLIR Tools software (*n* = 5 biological replicates).

### In vivo metabolic measurements

Mice were housed one per cage in the Oxy max/CLAMS metabolic cage system manufactured by Columbus Instruments (Columbus, OH, USA). Mice were acclimatized to the equipment and environment in the metabolic cages for one day prior to data collection. Subsequently, mice were monitored ad libitum for 72 h in cages at a temperature of 20–23 °C and a dark/light cycle of 12 h/12 h. Respiratory exchange ratio [V(O_2_)/V(CO_2_)] and heat were measured with the Oxy max system. Data from a stable 24-h period were selected for analysis (*n* = 4 biological replicates).

### Hematoxylin and eosin staining analysis

Briefly, the tissue was embedded first (*n* = 3 biological replicates). The embedded wax block was fixed on a slicer and sliced into thin slices of about 4 μm using a rotary microtome, dewaxing, to water, dyeing. Uterine morphology and number of glands were observed using a microscope (IX73; Olympus, Tokyo, Japan). The distance from the inner layer of the sow and mice endometrium to the myometrium was measured horizontally and vertically in the HE staining images of the three cross‐sections of the uterus, and the average value was calculated. The uterine collagen deposition was observed according to Masson's trichrome staining and analyzed by Image J software. The red area basically corresponds to the stained area of the uterine tissue, while the darker stained part of the blue area is the collagen fibre part, and the collagen fibre area and the uterine tissue area were obtained by setting the measurement parameters in Image J. Relative collagen area = collagen area/total tissue area × 100% (*n* = 4 biological replicates).

### Scanning electron microscopy

To assess the morphology of pinopodes, the uterus of 13-d gestation sows and 5.5-d gestation mice was incised to expose intrauterine structures. The surface was then rinsed with saline and placed in 2.5% glutaraldehyde for fixation. After fixation, the tissues were rinsed three times with PBS and fixed in 1% osmium tetroxide for 90 min then washed. Then, tissue blocks of 3 mm^2^ were dehydrated three times through a graded alcohol series and 100% acetone before being dried and plated with palladium for 30 s using an ion sputtering instrument (MSP-2S, IXRF, Austin, TX, USA). Three randomly selected areas of the endometrial epithelial surface of each sample were subjected to scanning micrographs, and the morphology & structure of pinopodes were analyzed with a scanning electron microscope (GeminiSEM 500; Zeiss, Germany) (*n* = 4 biological replicates).

### Embryo implantation, embryo development, and live births

Observed with vaginal plugs to localize pregnancy for 0.5 d, 6 female mice per group were anesthetized at 5.5 d, and 1% Evan’s blue dye (CAS No.: 2610-05-1 C8679, Sigma) dissolved in saline solution was injected tail vein. Mice were anesthetized and slaughtered at 5.5 dpc (*n* = 6 biological replicates), and uterine tissue was collected and the number of embryo attachments recorded. At 16.5 dpc (*n* = 7 biological replicates), embryos number was counted. Live births number was recorded on the third day after birth (*n* = 7 biological replicates).

### Statistical analysis

Statistical analysis was performed using SPSS 26.0 and GraphPad Prism 9.0. Two groups were analyzed using Student’s *t*-test, and 4 groups were analyzed using one-way ANOVA followed by Bonferroni’s test, with the level of significant difference set at *P* < 0.05. And the data pass the normal distribution test in Shapiro–Wilk test. Data are presented as mean ± SEM (standard error of the mean) and experiments were repeated at least 3 times.

## Results

### Uterine lipid accumulation, reduced endometrial receptivity, and lower levels of melatonin in obese sows

**Fig. 1:**
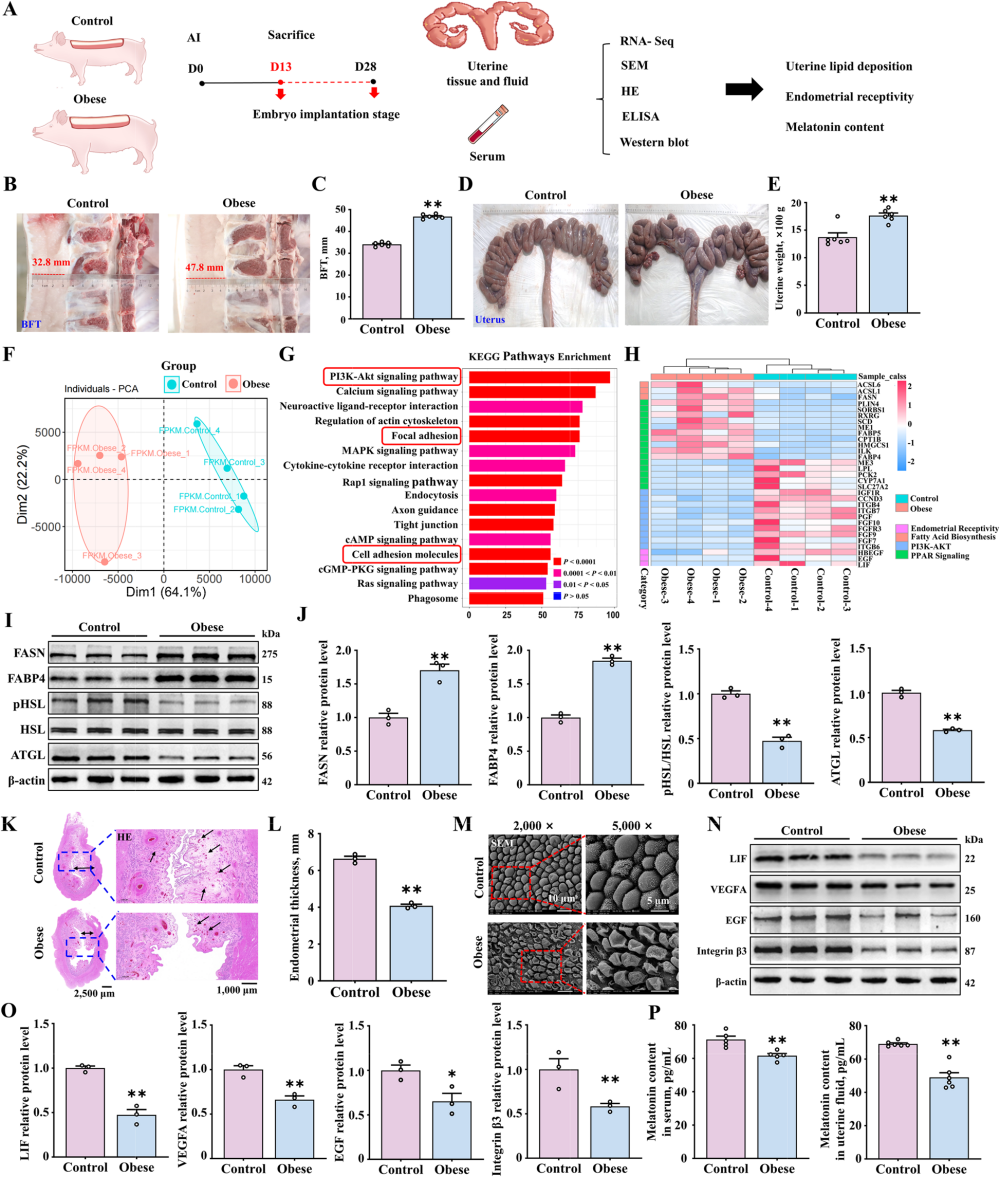
Increased lipid accumulation and decreased endometrial receptivity in high backfat thickness sows. **A** Uterine tissue and fluid and serum samples collected at 13 d after fertilization of the control and obese sows. **B**–**E** Backfat thickness and uterine weight in the control and obese groups (*n* = 6). **F**–**H** PCA, KEGG and Heat map data analysis of uterine RNA-seq data (*n* = 4). For KEGG Pathways Enrichment, we based on unadjusted *P*-values < 0.05. **I** and **J** The relative protein expression of FASN, FABP4, pHSL and ATGL (*n* = 3). **K** and **L** Uterine tissue samples stained with HE (scale bar = 2,500 μm/1,000 μm). The bi-directional black arrows indicate endometrial thickness, and the unidirectional black arrows indicate the glands (*n* = 3). **M** Pinopodes observed (scale bar = 10 μm/5 μm) (*n* = 3). **N** and **O** The relative protein expression of markers of endometrial receptivity (LIF, VEGFA, EGF, and Integrin β3) (*n* = 3). **P** Melatonin levels in the uterine fluid (*n* = 6) and serum samples (*n* = 5) of sows. ^*^P < 0.05; ^**^P < 0.01

We divided third-parity crossbred (Large White × Landrace) sows into two groups-the control and obese groups-according to visual Body Condition Scoring (BCS) [43]. Both groups underwent artificial insemination twice and were slaughtered on the 13^th^ d of insemination (that is, in the pre-implantation embryo stage). Following this, uterine tissue, luminal fluid, and serum samples were collected for analyses (Fig. 1A). Backfat thickness in the control group (*n* = 6) and the obese group (*n* = 6) was approximately 32.8 mm and 47.8 mm (Fig. 1B and C), respectively, and uterine weight in the obese group was significantly higher (Fig. 1D and E).

Comparison of RNA-seq data of uterine tissue from the control and obese pigs revealed that there were 2,031 upregulated and 2,622 downregulated genes (Fig. S1A). The samples from the two groups were well clustered, which is indicative of high reliability of the data (Fig. 1F, Fig. S1B). According to KEGG and GO analyses, the differentially expressed genes were mainly enriched in cell adhesion and migration processes related to embryo implantation, which reflect biological processes in the endometrium (Fig. 1G, Fig. S1C and D). Simultaneously, we performed enrichment analysis of genes related to endometrial receptivity, fatty acid synthesis, and the PPAR signaling pathway. The results confirmed that adipogenesis-related genes in the uterus were upregulated in the obese group, while the genes of *LIF*, *HB-EGF* and *EGF* were downregulated (Fig. 1H). Western blot results validated the RNA sequencing data and showed that the expression of the adipogenesis-related genes *FASN* and *FABP4* in the uterus of the obese group was significantly higher, while the lipolysis-related genes were significantly downregulated (Fig. 1I and J). These findings are suggestive of a possible negative correlation between the processes of adipogenesis and endometrial receptivity.

Endometrial structure was assessed by histological and scanning electron microscope analysis. Uterine tissue from obese sows showed significantly thinner endometrium and fewer glands compared to controls, and the pinopodes showed signs of indentation and collapse, with sparse microvilli. In the control group, endometrial thickness and number of glands were higher (Fig. 1K and L). Further, the pinopodes were intact, with dense and uniformly distributed microvilli (Fig. 1M). The uterus needs to enter a receptive phase for successful implantation. The protein levels of the LIF, EGF, and Integrin β3 in obese sows were significantly lower (Fig. 1N and O). Furthermore, we assessed the melatonin content in the serum and uterine cavity fluid of sows by ELISA and found that the melatonin content in the obese group was lower (Fig. 1P). Collectively, these results indicate that lipid accumulation in the uterus of obese sows might be responsible for decreased endometrial receptivity and lower melatonin content.

### Improvement in endometrial receptivity by lipid reduction in porcine endometrial epithelium cells treated with melatonin

**Fig. 2:**
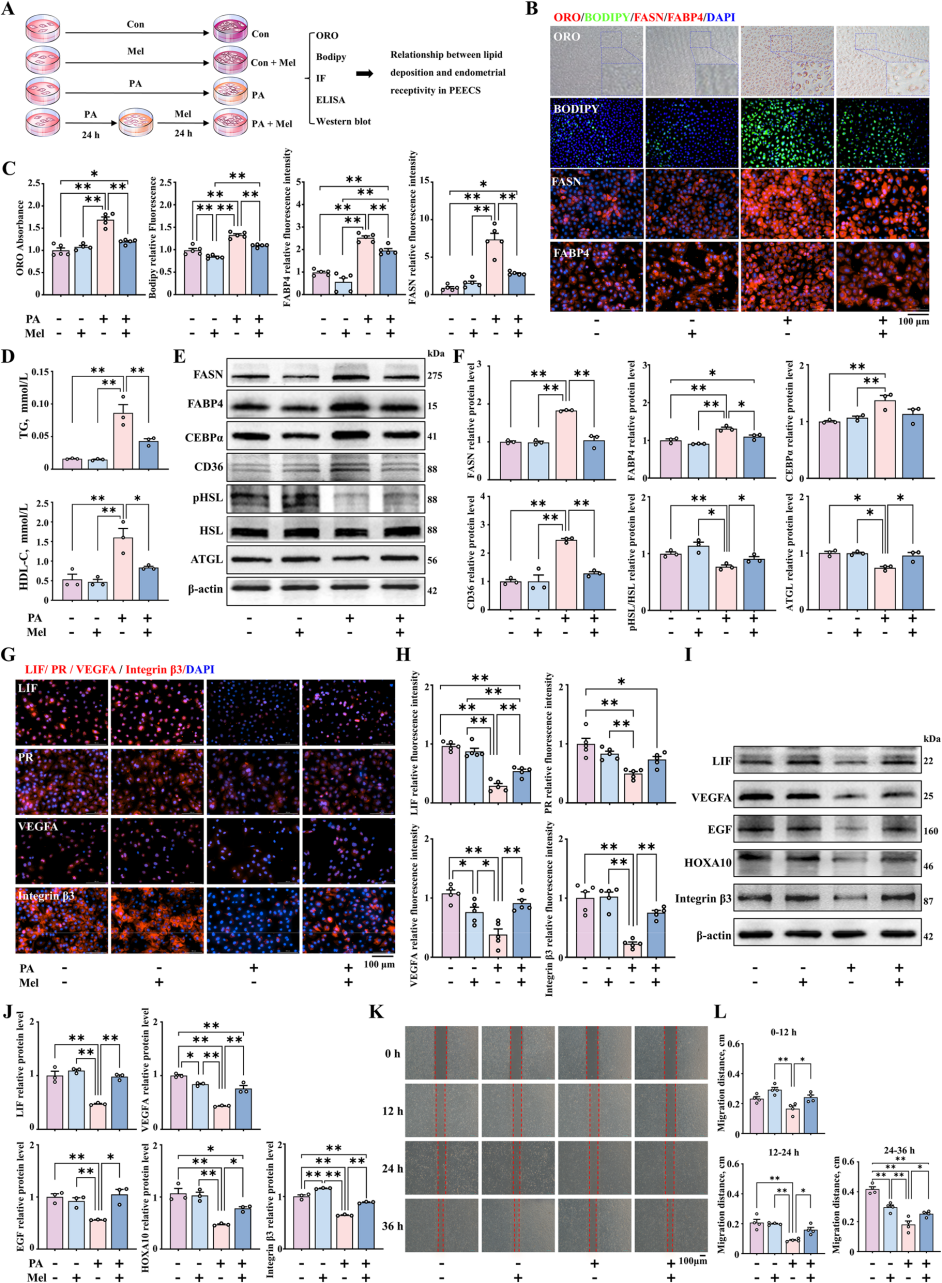
Improvement in endometrial receptivity with decrease in lipid deposition in PEECs. **A** Treatment of PEECs with PA and melatonin. **B** Oil Red O (*n* = 5), Bodipy staining (green) (*n* = 5), and immunofluorescence staining of FASN and FABP4 (red) (*n* = 5) (scale bar = 100 μm). **C** Quantification of cells stained with Oil Red O, Bodipy, FASN, and FABP4. **D** TG and HDL-C content measured in PEECs by ELISA (*n* = 3). **E** and **F** The protein expression of FASN, FABP4, CEBPα, CD36, pHSL and ATGL (*n* = 3). **G** Immunofluorescence staining of LIF, PR, VEGFA, and Integrin β3 (red) (scale bar = 100 μm) (*n* = 5). **H** Quantification of LIF, PR, VEGFA, and Integrin β3. **I** and **J** The relative protein expression of LIF, VEGFA, HOXA10, Integrin β3 (*n* = 3). **K** Representative bright-field images showing the migration of PEECs at 0, 12, 24, and 36 h (scale bar = 100 μm) (*n* = 4). **L** Quantitative results of wound closure rates. ^*^P < 0.05; ^**^P < 0.01. PA: palmitic acid, Mel: melatonin

To further illustrate the specific effect of uterine lipid deposition on endometrial receptivity, we established a uterine lipid deposition model by treating porcine endometrial epithelium cells (PEECs) with palmitic acid (PA) and investigated the role of melatonin under in vitro conditions of uterine lipid accumulation (Fig. 2A). We selected PA and melatonin doses of 40 µmol/L (Fig. S2B–D) and 20 µmol/L (Fig. S2G–I), respectively, for in vitro modeling because it is known that these doses do not affect the viability and proliferation of PEECs. This was confirmed by the Edu (Fig. S2B–C, G–H) and CCK-8 assays (Fig. S2D and I). Compared to the control group, PA led to a significant increase in lipid accumulation in PEECs, while co-treatment with melatonin significantly reduced lipid droplet area and size, as shown through Bodipy staining, Oil Red O staining and immunofluorescence staining of FASN and FABP4 (Fig. 2B and C). Similarly, we found that PA led to a significant increase in the intracellular levels of TG and HDL-C, both of which were significantly reduced after co-treatment with melatonin (Fig. 2D). Additionally, Western blot results indicated that PA led to a significant increase in the expression of the proteins of FASN, FABP4, CEBPα and CD36 (Fig. 2E and F, Fig. S2E and F), while the expression of the proteins pHSL and ATGL was decreased. After co-treatment with melatonin, the expression of the adipogenesis genes was downregulated (Fig. 2E and F, Fig. S2J and K). The above results reveal that melatonin can reduce PA-induced lipid deposition in PEECs.

Based on the above model, we further investigated whether lipid deposition in PEECs affects endometrial receptivity and assessed the impact of melatonin supplementation. Our result shown that abnormal lipid accumulation in the endometrium led to decreased endometrial receptivity. Presence of LIF, PR, VEGFA and Integrin β3 in PEEC determined by immunofluorescence results (Fig. 2G and H). Further, alleviation of lipid deposition through melatonin treatment was found to promote the expression of LIF, PR, VEGFA and Integrin β3, as demonstrated by Western blot (Fig. 2G–J). Endometrial cell migration is required for the pre-implantation stage. Hence, we also evaluated the impact of abnormal lipid accumulation in the uterus on cell migration rates. Wound healing assays showed that, the PA group exhibited significantly reduced cell migration and proliferation rates at 0, 12, 24, and 36 h, which improved after lipid accumulation was reduced with melatonin treatment (Fig. 2K and L). The above results collectively indicate that abnormal lipid accumulation in endometrial cells leads to decreased endometrial receptivity, which can be alleviated by melatonin.

### Role of the melatonin receptor MT2 in enhancing endometrial receptivity under uterine lipid accumulation

**Fig. 3:**
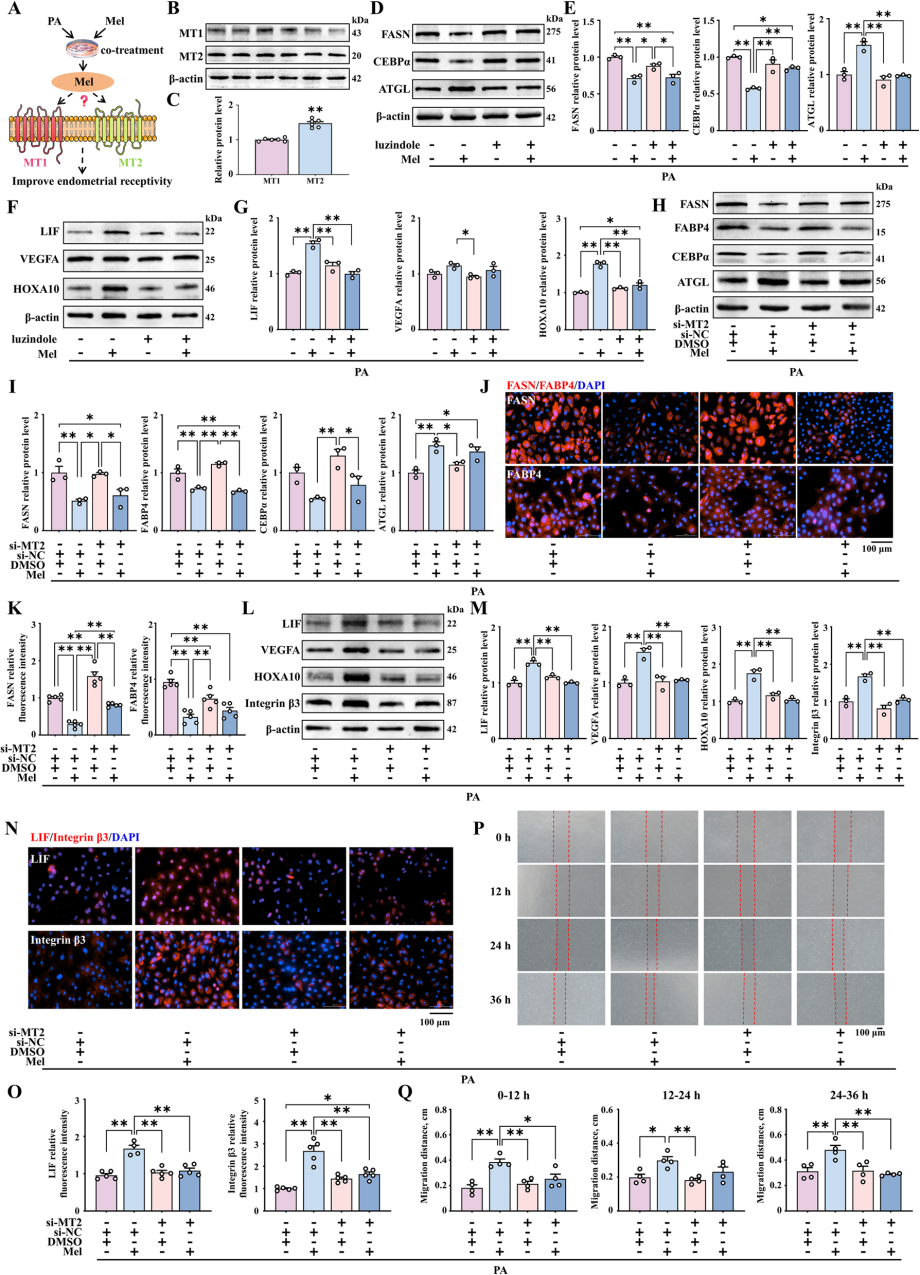
Enhanced endometrial receptivity after co-treatment with melatonin related to its specific binding to the MT2 receptor. **A** Modeling of how melatonin works in combination with MT1 or MT2. **B** and **C** The protein expression of MT1 and MT2 (*n* = 6). **D** and **E** The relative protein expression of FASN, CEBPα and ATGL (*n* = 3). **F** and **G** The protein expression of LIF, VEGFA, and HOXA10 (*n* = 3). **H** and **I** The protein expression of FASN, FABP4, CEBPα and ATGL (*n* = 3). **J** Immunofluorescence staining of FASN and FABP4 (red) (scale bar = 100 μm) (*n* = 4). **K** Quantification of FASN and FABP4. **L** and **M** The relative protein expression of LIF, VEGFA, HOXA10, and Integrin β3 (*n* = 3). **N** Immunofluorescence staining of LIF and Integrin β3 (red) (scale bar = 100 μm) (*n* = 4). **O** Quantification of LIF and Integrin β3. **P** Representative bright-field images showing the migration of PEECs at 0, 12, 24, and 36 h (scale bar = 100 μm) (*n* = 4). **Q** Quantitative results of wound closure rate. ^*^P < 0.05; ^**^P < 0.01

In sows, expression of the melatonin receptors 1A (MT1) and 1B (MT2) was increased in the uterine luminal epithelium as the pregnancy progressed in the early stage. Based on this observation, we further determined the contribution of the melatonin receptors to enhancing endometrial receptivity under the lipid deposition condition in PEECs (Fig. 3A). We first verified that MT1 and MT2 are expressed in PEECs (Fig. 3B and C). Luzindole, as an inhibitor of both MT1 and MT2, has been shown to effectively reduce the expression of MT1 and MT2 at a concentration of 500 nmol/L (Fig. S3A and B). In cells models that were treated with luzindole, melatonin did not reduce lipid accumulation in endometrial cells (Fig. 3D and E) or improve endometrial receptivity under conditions of obesity (Fig. 3F and G).

We further evaluated whether melatonin exerts its effects specifically through a single receptor or through the combined action of MT1 and MT2 by siRNA interference experiments (Fig. S3C and D). Western blot demonstrated that after si-MT2 treatment, melatonin could still reduce lipid deposition in endometrial cells (Fig. 3H and I). Meanwhile, immunofluorescence results were consistent (Fig. 3J and K). But it could not promote the expression of genes associated with endometrial receptivity (Fig. 3L–O). Similarly, the wound healing assay also confirmed that co-treatment with si-MT2 and melatonin does not enhance the cell migration rate at 12, 24, and 36 h (Fig. 3P and Q). These findings validate the role melatonin binding with MT2 in improving endometrial receptivity.

The Western blotting results indicated that inhibiting MT1 in PEECs treated with melatonin still results in reduced lipid deposition in endometrial cells (Fig. S4A and B). Immunofluorescence results are similar to protein results (Fig. S4C and D). And increases the expression of LIF, VEGFA, HOXA10 and Integrin β3 (Fig. S4E–H). In addition, the wound healing experiments also showed that melatonin can improve the migration rate of PEECs even after treatment with PA and siRNA against MT1 (Fig. S4I and J). To explore the potential reasons between the differences observed with inhibition of the MT1 and MT2 receptors, we also assessed their expression levels. Our results showed that the expression level of MT2 was significantly higher than that of MT1 (Fig. 3B and C). To sum up, our results imply that the role of melatonin in improving endometrial receptivity under conditions of uterine lipid accumulation is related to its specific binding to the MT2 receptor.

### Role of the PI3K/AKT signaling pathway in the inhibitory effect of melatonin on uterine lipid accumulation and the promotion of endometrial receptivity

**Fig. 4:**
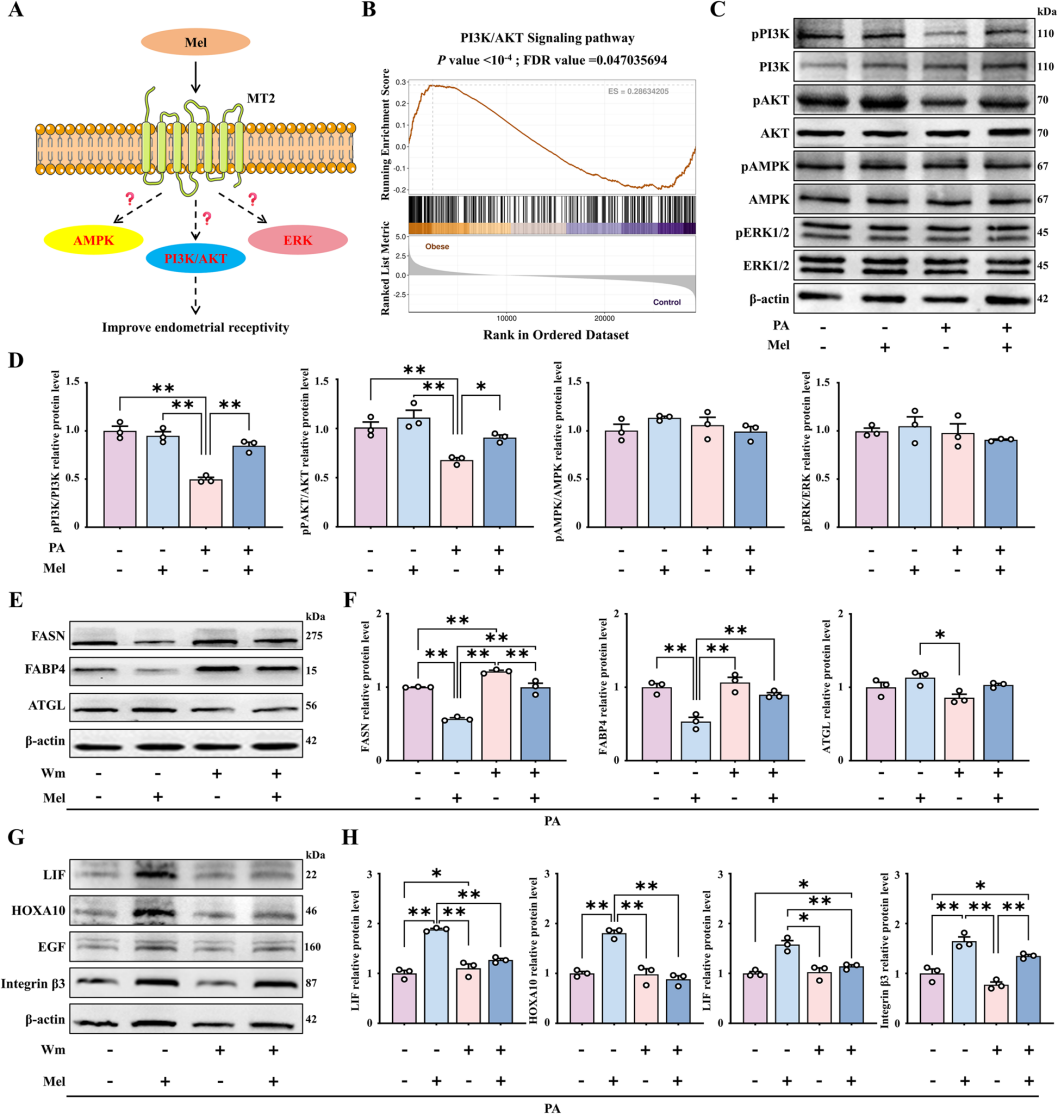
Role of PI3K/AKT activation in reducing uterine lipid deposition and improving endometrial receptivity. **A** Model diagram of the pathways involved in enhancing endometrial receptivity after melatonin binding to MT2. **B** RNA-seq analysis data showing the activation of the PI3K/AKT signaling pathway (*n* = 4). **C** and **D** The relative protein expression of pPI3K, PI3K, pAKT, AKT, pAMPK, AMPK, pERK, and ERK (*n* = 3). **E** and **F** The relative protein expression of FASN, FABP4 and ATGL. **G** and **H** The protein expression of LIF, HOXA10, EGF and Integrin β3 (*n* = 3). ^*^P < 0.05; ^**^P < 0.01, Wm: Wortmannin

Based on the above results, we further explored how the binding of melatonin to MT2 enhances endometrial receptivity in the lipid-rich uterus (Fig. 4A). RNA-seq analysis shown that differential genes were significantly enriched in the PI3K/AKT signaling pathway between obese and control sow uterine tissues (Fig. 1G, 4B, and Fig. S1E). We also validated the effects of melatonin on the metabolism-related pathways PI3K/AKT, AMPK and ERK by Western blot. It was found that PI3K/AKT signaling in endometrial cells was inhibited after PA treatment, further, melatonin effectively activated the PI3K/AKT signaling axis, and not any other pathway (Fig. 4C and D).

To further investigate the role of the PI3K/AKT axis in the melatonin-mediated reduction of lipid deposition in the uterus and improvement of endometrial receptivity, the PI3K inhibitor wortmannin was used to co-treat cells at a concentration of 100 nmol/L (Fig. S5A and B). As expected, PI3K knockout in PEECs reduced the effectiveness of melatonin in reducing lipid accumulation (Fig. 4E and F). Furthermore, the result shown that the endometrial receptivity-related proteins were also not significantly upregulated on wortmannin co-treatment (Fig. 4G and H). The findings imply that endometrial receptivity in obese animals may be reduced as a result of the inhibition of the PI3K/AKT signaling axis.

### Enhanced endometrial receptivity in obese animals treated with melatonin through activation of LIF/STAT3 signaling

**Fig. 5:**
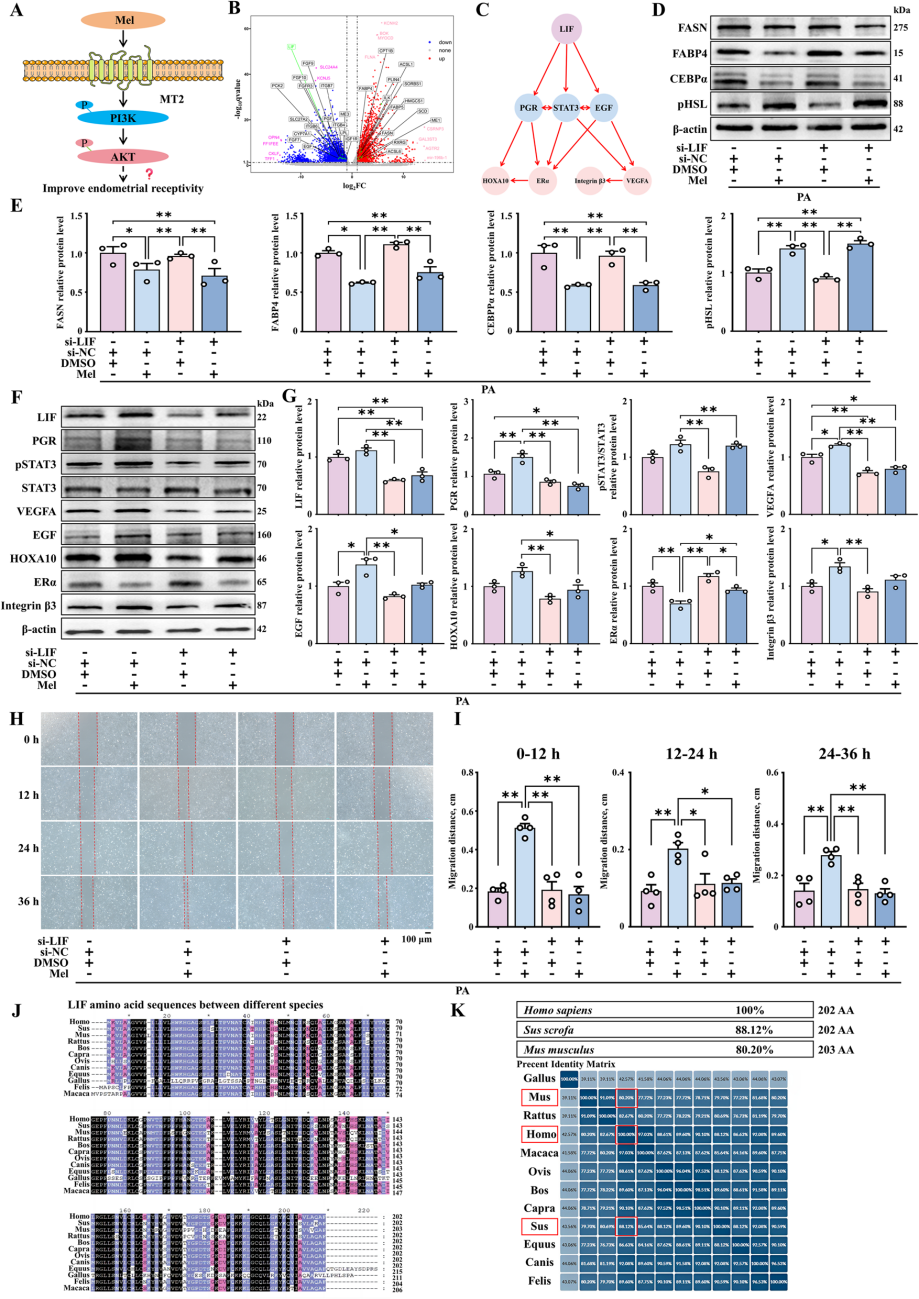
Role of LIF/STAT3 in the melatonin-mediated improvement in endometrial receptivity in obese animals. **A** Model map showing the role of PI3K/AKT in enhancing endometrial receptivity, with target gene prediction. **B** Volcanic map of lipogenic genes and endometrial receptivity genes. For volcano plot, we used adjusted *P*-values to control the false discovery rate (FDR). Genes with a −log_10_(q-value) greater than 1.3. **C** Endometrial receptivity gene and STAT3 protein interaction network. **D** and **E** The relative protein expression of FASN, FABP4, CEBPα and pHSL (*n* = 3). **F** and **G** The relative protein expression of LIF, PGR, VEGFA, EGF, HOXA10, Erα and Integrin β3 and pSTAT3/ STAT3 (*n* = 3). **H** Representative bright-field images of PEECs at 0, 12, 24, and 36 h after treatment (scale bar = 100 μm) (*n* = 4). **I** Quantitative results of wound closure rates. **J** and **K** LIF amino acid sequences between different species. ^*^P < 0.05; ^**^P < 0.01

To identify the potential endometrial receptivity-related genes regulated by the PI3K/AKT axis, we further screened the differentially expressed genes identified in RNA-seq (Fig. 5A). Among the significant genes related to adipogenesis and endometrial receptivity, LIF was identified as a potential target for PI3K/AKT based on its significant downregulation in obese sows (Fig. 1H and Fig. 5B). We also uploaded the data related to the endometrial receptivity-related genes *LIF*, *EGF*, and *VEGFA* to the STRING database (http://cn.string-db.org) to map protein network interactions. We then screened and beautified the gene interaction network obtained from the STRING online database using the Cytoscape software. Through the protein interaction network, we deduced that LIF may interact with related receptivity genes via STAT3 (Fig. 5C). Previous studies have demonstrated the important role of STAT3 in regulating epithelial remodeling and maintaining normal pregnancy during pregnancy [51].

To confirm the above observations, we established LIF knockout in PEECs by transfecting them with si-LIF (Fig. S6A). Western blot analysis of these cells showed that melatonin can still reduce lipid accumulation (Fig. 5D and E). But cannot improve endometrial receptivity in LIF-knockout PEECs (Fig. 5F and G). Similarly, si-LIF transfection affected the ability of melatonin to improve the migration rate of PEECs after co-treatment with melatonin (Fig. 5H and I). The above results revealed that LIF is a key target of PI3/AKT-mediated improvement in endometrial receptivity. Protein sequences of mouse, pig, and human genes were downloaded from the Uniport website for homology analysis, and sequence alignment and beautification were performed using the MEGA and GeneDoc software (Fig. 5J and K). Next, we validated and analyzed the above results in a mouse model.

### Maintenance of metabolic balance and reduction of lipid deposition in HFD mice treated with melatonin

**Fig. 6:**
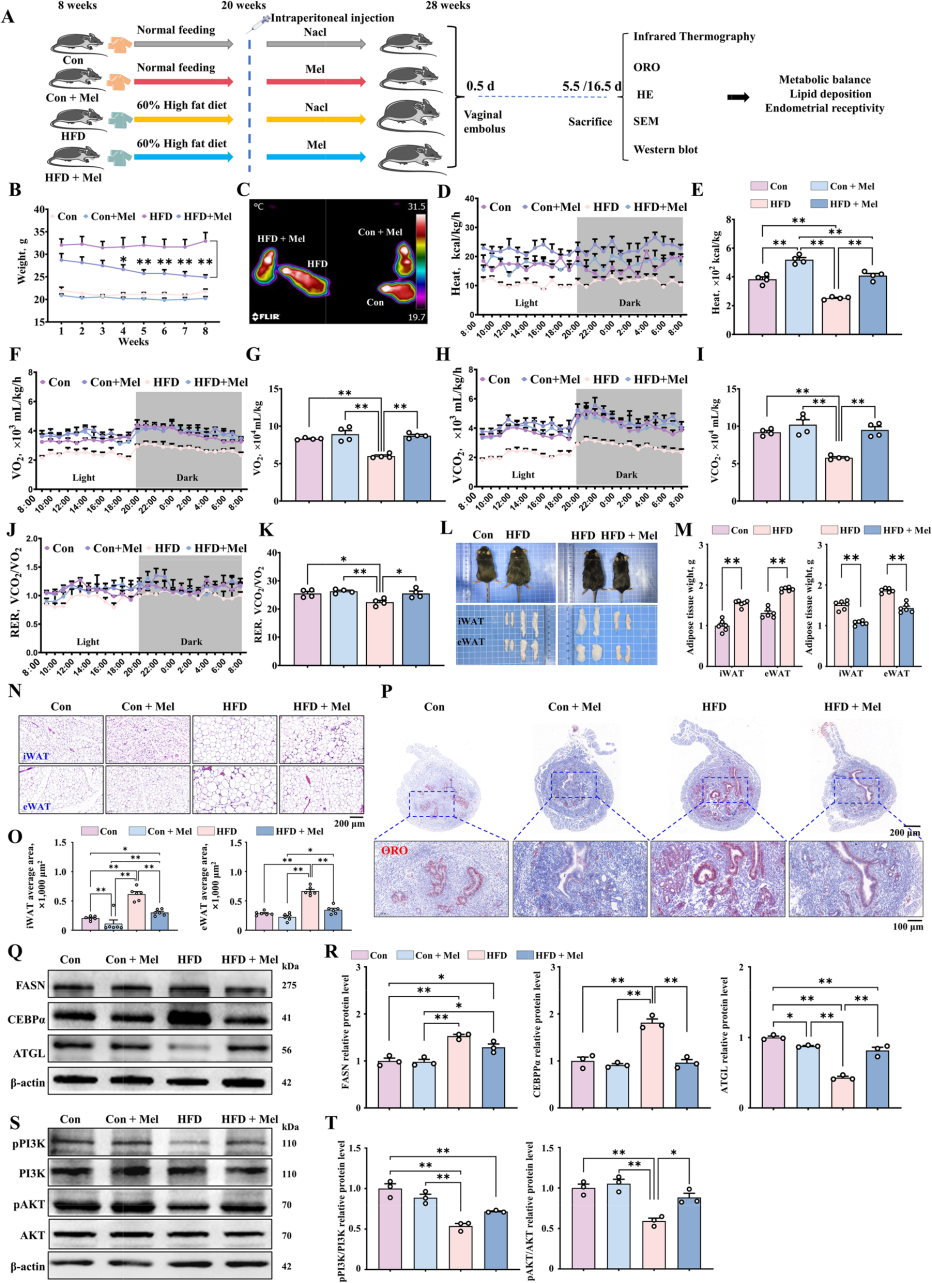
Alleviation of metabolic disorders caused by obesity and abnormal lipid deposition by melatonin treatment. **A** Modeling and treatment diagram of the mouse model. **B** Body weight change in mice (*n* = 20). **C**–**E** Heat production and body temperature change in mice (*n* = 4). **F**–**K** Carbon dioxide exhaled, oxygen consumption, and respiratory quotient in the mice. **L** and **M** iWAT and eWAT morphology and weight in different groups (*n* = 4). **N** iWAT and eWAT tissue samples from each group with HE staining (scale bar = 200 μm) (*n* = 6). **O** Lipid droplet area of iWAT and eWAT samples (*n* = 6). **P** Oil Red O staining of uterine tissue (scale bar = 500 μm/100 μm) (*n* = 4). **Q** and **R** The relative protein expression of FASN, CEBPα and ATGL (*n* = 3). **S** and **T** The relative protein expression of pPI3K, PI3K, pAKT, and AKT. ^*^P < 0.05; ^**^P < 0.01

To investigate whether melatonin can be used to improve metabolic capability in HFD mice, 20 mg/kg melatonin was injected intraperitoneally for up to 8 weeks (Fig. 6A). The dose of 20 mg/kg body weight melatonin has been widely used and is approximately equivalent to a human dose of 3.24 mg/kg body weight of body weight, that is, about 200 mg for a person weighing 60 kg, which has been shown to be safe [52, 53]. Significant weight loss was observed even with fairly consistent food intake after melatonin administration in mice (Fig. 6B). Moreover, melatonin was found to maintain metabolic balance in HFD mice based Infrared thermography (Fig. 6C) and metabolic cage experiments (Fig. 6D and E). The main manifestations are increased oxygen consumption (Fig. 6F and G), increased carbon dioxide exhalation (Fig. 6H and I) and increased respiratory quotient (Fig. 6J and K). In addition, the weights of iWAT and eWAT (Fig. 6L and M) and lipid droplet area were reduced in slaughtered mice at 5.5 d of pregnancy (Fig. 6N and O). As expected, melatonin significantly reduced lipid deposition in the uterus (Fig. 6P). This observation was consistent with the decrease in the protein expression of lipid deposition-related genes in the uterus (Fig. 6Q and R).

Next, we verified the mechanism by which melatonin enhances receptivity in HFD mice. The Western blot results showed that obesity can inhibit the PI3K/AKT pathway, and melatonin can significantly increase the activity of this pathway (Fig. 6S and T). The above results indicate that melatonin maintains metabolic balance and reduces uterus lipid deposition in HFD mice by activating the PI3K/AKT signal axis.

### Increase in endometrial receptivity and embryo implantation rate in HFD mice treated with melatonin

**Fig. 7:**
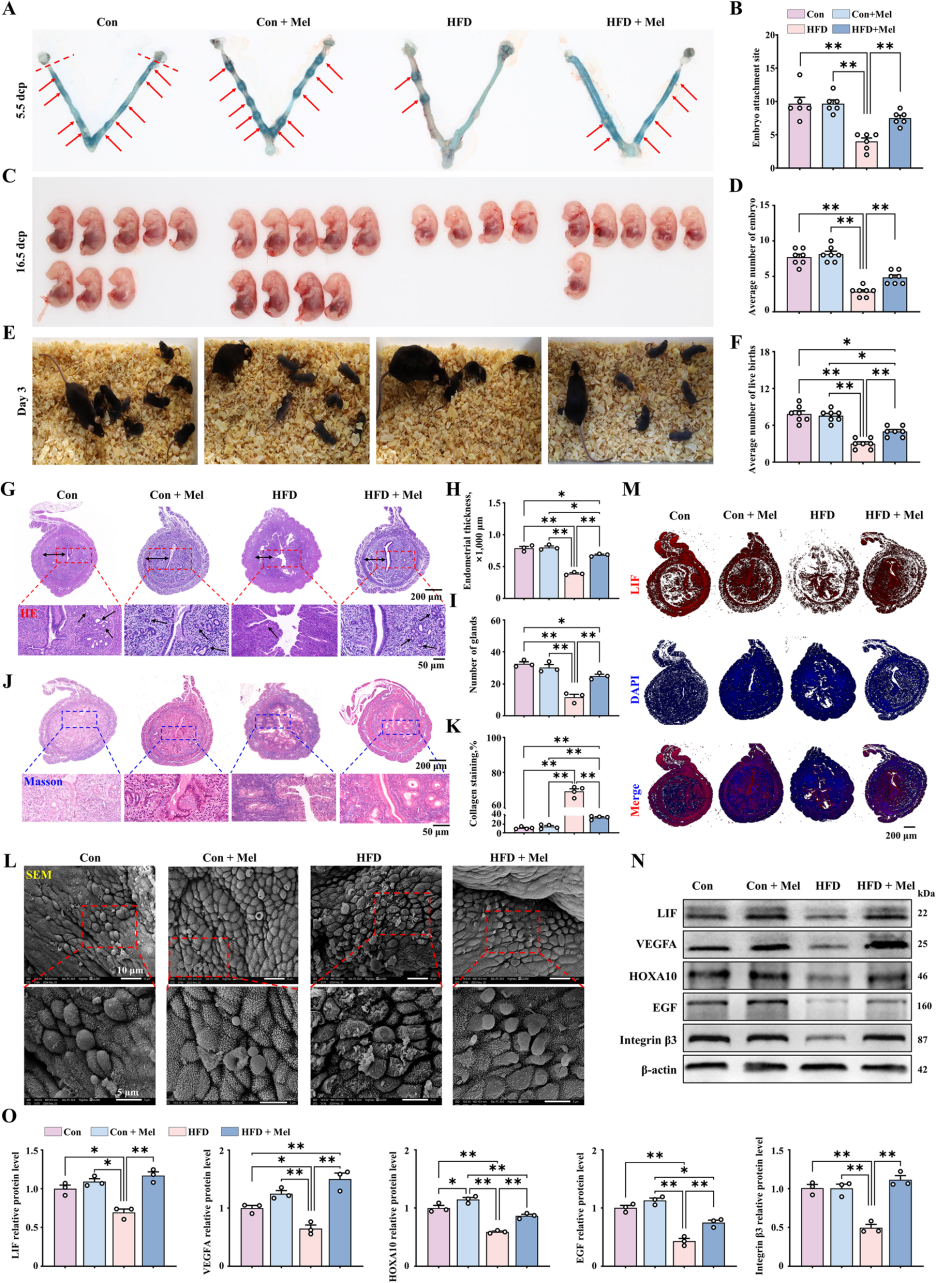
Increase in endometrial receptivity and implantation rate in obese mice treated with melatonin. **A** Embryo implantation (*n* = 6). **B** Average number of embryo implantation sites. **C** and **D** Embryonic development in pregnant mice (*n* = 7). **E** Number of live births of pregnant mice (*n* = 7). **F** Average number of live births. **G** Uterine tissue samples stained with HE (scale bar = 50 μm/200 μm). The bi-directional black arrows indicate endometrial thickness, and the unidirectional black arrows indicate the glands. **H** Endometrial thicknesses in each group (*n* = 3). **I** Number of glands in each group. **J** Masson’s trichrome staining for evaluating endometrial fibrosis (blue is indicative of scar formation) (scale bar = 50 μm/200 μm). **K** Quantification of collagen protein expression levels (*n* = 3). **L** Pinopodes observed under a scanning electron microscope. **M** Immunofluorescence staining of LIF in uterine tissue (red) (scale bar = 200 μm) (*n* = 3). **N** and **O** The relative protein expression of LIF, VEGFA, HOXA10, EGF, and Integrin β3 (*n* = 3). ^*^P < 0.05; ^**^P < 0.01

To further explore whether melatonin improves embryo implantation in obese animal, we slaughtered impregnated mice at 5.5 d of gestation. The results showed that the number of embryos implanted was significantly lower in HFD mice. Melatonin injection significantly increased in the number of implanted embryos compared to the HFD mice that did not receive melatonin (Fig. 7A and B). To further explore embryonic development and litter size, we counted the number of embryos at 16.5 d and offspring at 3 d of birth and found that they were significantly lower in the HFD group. However, significantly higher numbers of embryos (Fig. 7C and D) and offspring (Fig. 7E and F) were observed in the HFD mice treated with melatonin. At 5.5 d of pregnancy, mice in the HFD group had incomplete uterine tissue with significant thinning of the endometrium and few glands. Melatonin treatment was found to effectively alleviate the shedding of the uterine glands (Fig. 7G–I). We stained the mouse uterus with Masson's stain, in order to observe collagen deposition and endometrial fibers. Endometrial collagen deposition is significantly increased in the uterus of HFD mice, while in the HFD group with melatonin treatment, the endometrial collagen deposition almost recovered to a level comparable to that of the control group (Fig. 7J and K). Observation of pinopodes on the endometrial tissues of the four mouse groups through scanning electron microscope showed that the endometrial pinopode structure of the uterus was collapsed and the microvilli were reduced in the HFD mice. However, in the HFD group treated with melatonin, the structure of the pinopodes was smooth, with an increased amounts of cilia that were also uniform (Fig. 7L).

Moreover, the percentage of LIF positivity in the endometrial epithelium of HFD mice was significantly reduced, while their numbers were close to those of the normal mice in the HFD mice treated with melatonin (Fig. 7M). Evaluation of the expression of endometrial receptivity-associated genes showed that significantly lower protein levels of LIF, VEGFA, and HOXA10 in the HFD mice than normal group (Fig. 7N and O), but the levels were considerably recovered in the HFD mice treated with melatonin (Fig. 7M–O). Together, these findings reveal the effectiveness of melatonin in improving endometrial receptivity and embryo number in obese animals.

## Discussion

**Fig. 8:**
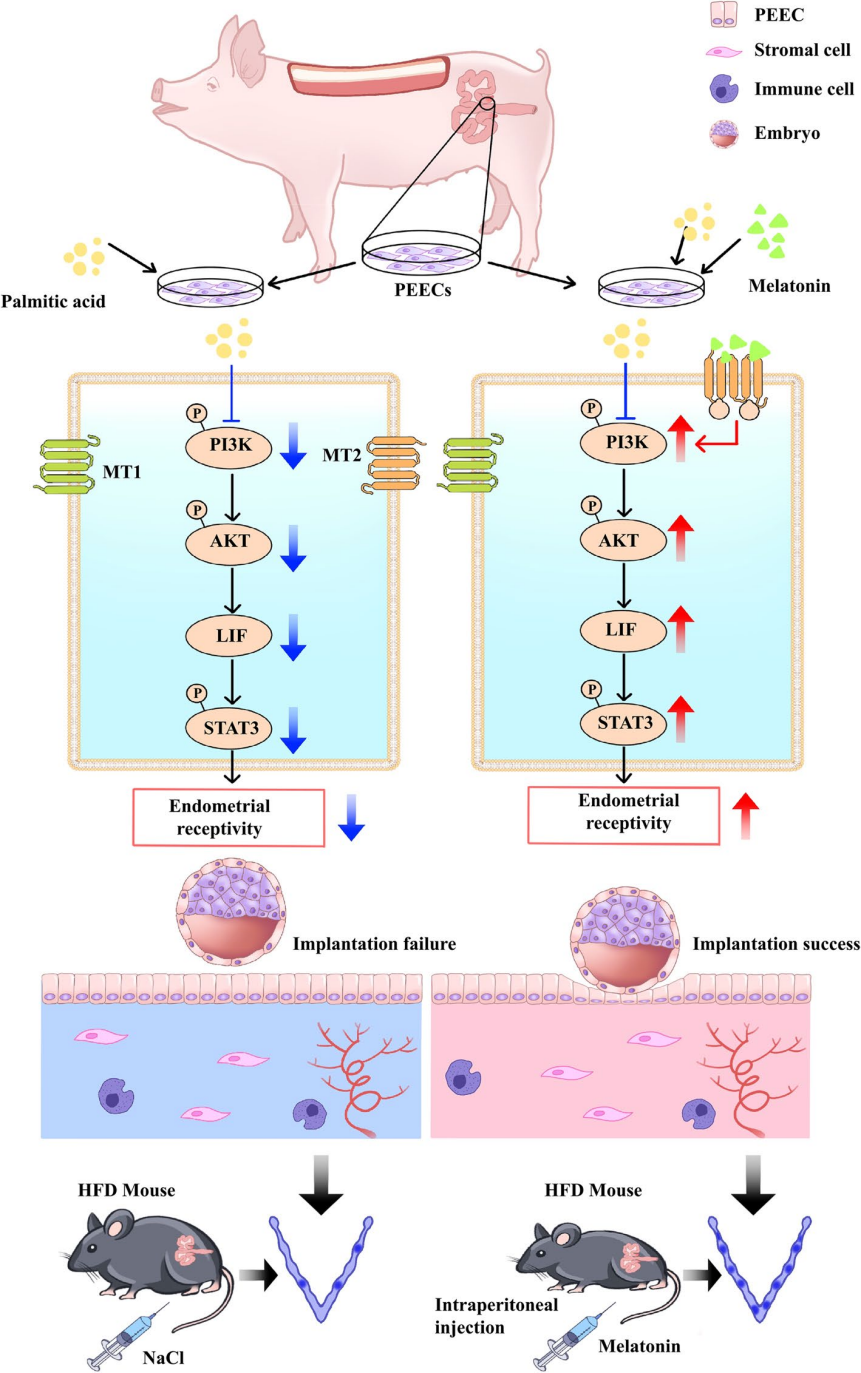
Schematic model depicting the effect of melatonin on endometrial receptivity and embryo implantation in obese animals. In both the in vitro model of PA-induced lipid formation in PEECs and the in vivo model of obese sow and HFD mice, inhibition of the PI3K/AKT/LIF/STAT3 signaling pathway was observed in uterine tissue, along with reduced endometrial receptivity and number of embryo implantation. The data demonstrate the benefits of melatonin treatment in obese animals, and the MT2/PI3K/AKT/LIF/STAT3 axis in implicated in the melatonin-mediated increase in endometrial receptivity and embryo implantation rate

In this study, we found that high backfat thickness sows are detrimental to embryo implantation by examining endometrial receptivity and melatonin levels, as well as the pathways that may be involved, in vitro cellular and in vivo animal models of obesity. In addition, we analyzed the effect of melatonin treatment and found that it reversed the decrease in endometrial receptivity induced by obesity. None of the previous studies on the effect of melatonin have focused on uterine lipid accumulation, so we believe that our study is the first to show that melatonin can also reduce lipid deposition in the uterus. Further, as shown in Fig. 8, we found that the MT2-PI3K-LIF axis plays a role in the improvement in endometrial receptivity and embryo implantation in obese animals after melatonin treatment.

In the sow, backfat thickness was found to be closely associated with lipid accumulation in the uterus and low melatonin levels, which (in turn) were related with decreased endometrial receptivity. Backfat thickness is an intuitive, easy to measure, and relatively stable index that reflects the body fat reserve of sows at different gestation stages. In line with our findings, it is closely related to the reproductive performance of sows and is, therefore, widely used to evaluate the body condition of sows and predict their reproductive performance [54]. Endometrial morphology and thickness have been shown to be related to endometrial receptivity. Here, we found that the thickness of the uterus in obese sows and HFD mice was decreased. Another structure that is also an important indicator of endometrial receptivity is the pinopode, which is a mushroom-like protrusion located on the luminal epithelium that can be observed under an electron microscope. During the window of implantation, the number and density of pinopodes rapidly increases in readiness for embryo implantation [55, 56]. We observed a decrease in the density of pinopodes and shedding of microvilli in excessively backfat thickness sows and HFD mice. As indicated above, these changes may be indicators of decreased endometrial receptivity in obese animals.

A large amount of fat deposition in the uterus may result from increased fat production in response to continuous high-energy feeding. This may affect blood circulation in the uterine wall, affecting uterine function and embryonic development [57, 58]. We also evaluated the levels of FASN and FABP4, and showed that FASN and FABP4 expression was significantly increase in the uterus of high backfat sows and HFD mice. We suspect that the adipose tissue in obese animal may produce factors, such as free fatty acids that cause metabolic disorder, thereby ultimately affecting the function of the endometrium. Because, accumulation of fatty acids may weaken endometrial function [59]. In line with these notions, previous research has also shown that the metabolic disruption associated with obesity leads to endometrial dysfunction [1]. Therefore, this study implies that decreased metabolic levels due to obesity are associated with decreased endometrial receptivity.

According to our RNA-Seq results, PI3K/AKT axis showed the most significant difference between obese and normal sows. This result is consistent with previous findings that the PI3K/AKT signaling axis is the major signaling pathway responsible for the differences between normal weight PCOS and overweight/obese PCOS women [60]. Thus, the differences in endometrial receptivity observed between the obese and control animals could be attributable to differences in uterine metabolic patterns mediated by the PI3K/AKT pathway.

Our results have demonstrated low melatonin levels in the uterine fluid and serum of obese sows and HFD mice. Low levels of melatonin are related to embryo attachment rates and difficulties in maintaining pregnancy [60, 61] and may explain the decreased embryo implantation sites in the obese animals in this study. We also investigated whether melatonin supplementation improves embryo implantation in mice associated with obesity and ultimately influence endometrial receptivity by administering it to the HFD mice at a daily dose of 20 mg/kg body weight for 8 weeks, based on the dose reported in previous research [62, 63]. Our results showed that melatonin administration reduces total body fat, but more specifically, uterus fat in HFD mice. Consistent with these in vivo findings, melatonin treatment at a dose of 20 µmol/L was found to significantly reduce lipid accumulation in PEECs. This effect of melatonin can be explained by its biological effects adipose tissue metabolism, that is, lipolysis [40, 64]. It also has been demonstrated that melatonin induces lipolysis in rats, and this is further supported by the diminished lipolysis in rats after pinealectomy [65]. In this study, the endometrium of the mice appeared thicker and the number of uterine glands was higher after melatonin treatment compared to the endometrium in untreated HFD mice. In addition, melatonin could effectively reduce collagen deposition, thereby promoting the recovery of reproduction and efficient live births in HFD mice. Further, endometrial receptivity was improved, the number of embryo attachment sites had increased, and the litter size was also increased. Previous studies have also shown that daily administration of melatonin to rats restores optimal implantation rates and improves uterine function in adult rats [59, 66]. Importantly, melatonin treatment did not affect the development and growth of pups at later stages. Thus, melatonin seems to improve endometrial receptivity and embryo implantation by enhancing the metabolic level and reducing uterine lipid deposition in obese animals.

Melatonin normally acts on two receptors, MT1 and MT2, which are found in the reproductive tissues of many mammals, such as the uterus [41]. To explore the signal axis via which melatonin regulates embryo implantation, we evaluated the protein levels of MT1 and MT2 in PEECs and uterine at 5.5 d of pregnancy, and the results showed that both receptors were expressed but had different expression levels. Further, similar to previous studies, the expression of both receptors increased over time, especially in the trophectoderm and uterine luminal epithelium during the early stages of pregnancy [67]. Notably, we found that the expression of MT2 is higher than that of MT1 in the endometrium; this could mean that MT2 is the dominant receptor involved in the effects of melatonin on reproduction. Thus, melatonin may induce lipolysis and increase the levels of receptivity-related genes to facilitate implantation by binding specifically to MT2. Similarly, previous studies have reported that melatonin could modulate LIF and facilitate implantation by binding specifically to MT2 [41]. Further, another study showed that melatonin binding to MT2 in the uterus of pre-pregnant mice increased the implantation rate and litter size [68]. In addition, we found that the main metabolic pathway enriched in the uterus is the PI3K/AKT axis which is mainly mediated by MT2. Thus, the different effects of melatonin binding to MT1 and MT2 in reducing uterine lipid deposition and maintaining pregnancy may be due to the fact that the structure of MT1 and MT2 or the mechanism of recognizing ligands and warrants further exploration [69]. Importantly, the PI3K/AKT signaling axis responds to MT2 regulation, and this implies that it is the specific binding of melatonin to MT2, and not MT1, that improves endometrial receptivity in obese animals.

In the next set of experiments, we investigated the mechanism by which melatonin improve endometrial receptivity. We found that melatonin is essential for PI3K/AKT activity, and we also excluded other classical signaling pathways that may be involved in the metabolic functions of melatonin. The results show that melatonin stimulated endometrial cell interactions via activation of MT2 and caused an increase in embryo implantation-related genes via activation of the PI3K pathway. Moreover, co-treatment with the PI3K inhibitor wortmannin and melatonin caused a decrease in the expression of endometrial receptivity-related genes, as well as proliferation and migration of PEECs. This confirms our hypothesis that PI3K/AKT activation promotes endometrial receptivity in the uterus. Altogether, these results reveal that the PI3K/AKT axis is critical for melatonin-mediated reduction in lipid deposition in the uterus and improvement of endometrial receptivity in obese animals.

LIF is expressed in the endometrium immediately before the onset of implantation and is an important marker gene for endometrial receptivity [70–74]. As expected, RNA-seq results showed a significant decrease in the expression of LIF in the endometrium of obese sows. This is in line with studies where Metwally et al. observed a negative correlation between endometrial LIF concentration and BMI and may explain why overweight/obese women repeatedly experience unexplained miscarriages [74, 75]. Next, we suppressed the expression of LIF and found that melatonin did not improve the expression of VEGFA, HOXA10 and EGF in the obese state. In addition, the levels of pSTAT3 were markedly reduced, resulting in a phenotype related to LIF deficiency and, thus, supporting previous observations that upregulation of pSTAT3 is essential to regulate implantation [51, 73]. Therefore, the reduced expression of LIF in the endometrium and its association with excessive lipid accumulation may also be one of the mechanisms involved in the reduction in fertility in obese animals. The related mechanism needs to be investigated in more detail in the future.

In this study, lipid deposition and decreased melatonin content in the uterus of sow with high backfat thickness were found to be associated with a decrease in endometrial receptivity and embryo implantation rate, and the MT2/PI3K/LIF signaling pathway was associated with melatonin-mediated improvement in endometrial receptivity. Most importantly, we provide evidence in support of the functional roles and signal transduction pathways of melatonin in the regulation of embryo implantation during early pregnancy in obese animals. With regard to molecular mechanism exploration, a more comprehensive analysis of all melatonin-related mechanisms involved in reproductive function would be beneficial in the future.

## Conclusions

Overall, this study unveils a novel link between high backfat thickness in sows and metabolic dysfunction in the uterus, where abnormal lipid accumulation and significantly reduced melatonin levels collectively impair endometrial receptivity. To the best of our knowledge, this is the first study to demonstrate the association of low melatonin levels with uterine lipid deposition. Meanwhile, we highlight the MT2/PI3K/LIF signaling pathway as a crucial target for enhancing endometrial receptivity during early pregnancy in obese animals.

## Supplementary Information

Additional file 1: Table S1. Sequences of primers used for real-time polymerase chain reaction.

Additional file 2: Table S2. Antibodies used for this study.

Additional file 3: Fig. S1. Differential gene enrichment analysis with RNA-Seq. A Differentially expressed genes in two groups. B Uterine heat map. C–D GO enrichment. E Enrichment of the PI3K/AKT axis. ^**^P< 0.01*, q* < 0.05.

Additional file 4: Fig. S2. Determination of PA and melatonin concentrations in vitro PEEC model. A Diagram showing the different concentrations of PA and melatonin used to treat PEECs. B Immunofluorescence staining with EdU (red) in PEECs (scale bar = 100 μm). C EdU-positive rate in PEECs (*n* = 3). D Cell viability detection (*n* = 4). E–F Western blotting evaluation of the relative protein expression of markers of lipogenesis (FASN, FABP4, CEBPα, and CD36) and lipolysis (pHSL and ATGL) (*n* = 3). G Immunofluorescence staining of EdU (red) in PEECs (scale bar = 100 μm). H EdU-positive staining rate (*n* = 3). Cell viability of PEECs (*n* = 4). J–K Western blotting analysis of the relative protein expression of markers of lipogenesis (FASN, FABP4, CEBPα, and CD36) and lipolysis (pHSL and ATGL) (*n* = 3). ^*^P< 0.05; ^**^P < 0.01.

Additional file 5: Fig. S3. Concentration screening of MT1 and MT2 inhibitor luzindole. A–B The relative protein expression of MT1 and MT2 (*n* = 3). C Quantification of MT1 interference efficiency (*n* = 5). D Quantification of MT2 interference efficiency (*n* = 5). ^*^P < 0.05; ^**^P< 0.01.

Additional file 6: Fig. S4. No improvement in endometrial receptivity on melatonin binding to the MT1 receptor. A–B The relative protein expression of FASN, FABP4, and CEBPα and ATGL (*n* = 3). C Immunofluorescence staining of FASN and FABP4 (red) (scale bar = 100 μm). D Quantification of FASN and FABP4 expression (*n* = 4). E–F The relative protein expression of LIF, VEGFA, HOXA10 and Integrin β3 (*n* = 3). G Immunofluorescence staining of LIF and Integrin β3 (red) (scale bar = 100 μm). H Quantification of LIF and integrin β3 (*n* = 4). I Representative bright-field images showing the migration of PEECs at 0, 12, 24, and 36 h (scale bar = 100 μm). J Quantitative results of wound closure rates (*n* = 4). ^*^P < 0.05; ^**^P < 0.01.

Additional file 7: Fig. S5. Determination of the PI3K-specific inhibitor wortmannin. A–B The protein expression of pPI3K, PI3K, pAKT, and AKT in sows (*n* = 3). ^*^P< 0.05; ^**^P < 0.01.

Additional file 8: Fig. S6. Quantification of LIF interference efficiency (*n* = 8). ^*^P < 0.05; ^**^P < 0.01.

## Data Availability

All data measured or analyzed during this work are available from the corresponding author upon reasonable request.

## Abbreviations

AKT: Protein kinase B
ATGL: Adipose triglyceride lipase
CEBPα: CCATT enhancer-binding protein α
DAPI: 4′,6-Diamidino-2-phenylindole
DMEM: Dulbecco's Modified Eagle Medium
EGF: Epidermal growth factor
ERα: Estrogen receptor α
EDTA: Ethylenediamine tetraacetic acid
eWAT: Epididymal white adipose tissue
FABP4: Fatty acid binding protein 4
FASN: Fatty acid synthase
GO: Gene Ontology
HSL: Hormone-sensitive lipase
HOXA10: Homeobox protein Hox-A10
iWAT: Inguinal white adipose tissue
KEGG: Kyoto Encyclopedia of Genes and Genomes
LIF: Leukemia inhibitory factor
MT1: Melatonin receptor 1
MT2: Melatonin receptor 2
Mel: Melatonin
PA: Palmitic acid
PEEC: Porcine endometrial epithelial cell
PHSL: Phosphorylation hormone-sensitive lipase
PR: Progesterone receptor
pSTAT3: Phospho-STAT3
PI3K: Phosphatidylinositide 3-kinases
PBS: Phosphate buffer saline
pTr: Porcine trophectoderm
pLE: Endometrial luminal epithelium
TG: Triglyceride
VEGFA: Vascular endothelial growth factor A
WM: Wortmannin
