# Supplementary material for: Melatonin improves endometrial receptivity and embryo implantation via MT2/PI3K/LIF signaling pathway in sows
Source: J Anim Sci Biotechnol. 2025 Jan 4;16:4. doi: 10.1186/s40104-024-01137-x (PMC11699789; doi:10.1186/s40104-024-01137-x)
Supplement: Supplementary file 1 — Additional file 1: Table S1. Sequences of primers used for real-time polymerase chain reaction. [file 40104_2024_1137_MOESM1_ESM.docx]

**Table S1. Sequences of primers used for real-time polymerase chain reaction**

| Target | Sequence (5’→3’) | Accession | concentrations | base pairs |
| --- | --- | --- | --- | --- |
| GAPDH | Forward: AGGTCGGTGTGAACGGATTTG | [NM_001206359.1](https://www.ncbi.nlm.nih.gov/nuccore/NM_001206359.1) | 100μmol | 21bp |
|  | Reverse: TGTAGACCATGTAGTTGAGGTCA |  | 100μmol | 23bp |
| β-actin | Forward: CTGGCACCACACCTTCTACAA | NM_001164650.1 | 100μmol | 21bp |
|  | Reverse: GTGTTGAAGGTCTCGAACATGAT |  | 100μmol | 23bp |
| MT1 | Forward: TCCTGCAAGAAGAGCTGCTG | [NM_001001266.2](https://www.ncbi.nlm.nih.gov/nuccore/NM_001001266.2) | 100μmol | 20bp |
|  | Reverse: AGGGCTTCCCCAGACTTCA |  | 100μmol | 19bp |
| MT2 | Forward: CCATGGATCCCAACTGCTCC | [XM_003355808.4](https://www.ncbi.nlm.nih.gov/nuccore/XM_003355808.4) | 100μmol | 20bp |
|  | Reverse: GCAGGAGCAGCAGCTTTTCT |  | 100μmol | 20bp |
| LIF | Forward: ACCGCATCATCGCCTACCTT | [NM_214402.2](https://www.ncbi.nlm.nih.gov/nuccore/NM_214402.2) | 100μmol | 20bp |
|  | Reverse: GACCTGCTTATACTTCCCCA |  | 100μmol | 20bp |
