# Supplementary material for: Melatonin improves endometrial receptivity and embryo implantation via MT2/PI3K/LIF signaling pathway in sows
Source: J Anim Sci Biotechnol. 2025 Jan 4;16:4. doi: 10.1186/s40104-024-01137-x (PMC11699789; doi:10.1186/s40104-024-01137-x)
Supplement: Supplementary file 2 — Additional file 2: Table S2. Antibodies used for this study. [file 40104_2024_1137_MOESM2_ESM.docx]

Table S2. Antibodies used for this study.

| Antibody | Company | Catalog | Dilution ratio | Species reactivity |
| --- | --- | --- | --- | --- |
| ATGL | Cell Signaling Technology | 2138 | 1:1000(WB) | rabbit |
| HSL | Cell Signaling Technology | 4107 | 1:1000(WB) | rabbit |
| FABP4 | Cell Signaling Technology | 2120 | 1:1000(WB) /1:100(IF) | rabbit |
| FASN | Cell Signaling Technology | 3180 | 1:1000(WB) /1:100(IF) | rabbit |
| CEBPα | Cell Signaling Technology | 2295 | 1:1000(WB) | rabbit |
| VEGFA | Affinity-Biosciences | AF5131 | 1:1000(WB)/1:100(IF) | rabbit |
| PR | Affinity-Biosciences | AF6106 | 1:100(IF) | rabbit |
| MT1 | Affinity-Biosciences | DF13877 | 1:1000(WB) | rabbit |
| MT2A | Affinity-Biosciences | DF6755 | 1:1000(WB) | rabbit |
| LIF | Proteintech | 26757-1-Ap | 1:1000(WB)/1:100(IF) | rabbit |
| HOXA10 | Proteintech | 26497-1- Ap | 1:1000(WB) | rabbit |
| Integrin beta 3 | Huabio | ET1606-49 | 1:1000(WB)/1:100(IF) | rabbit |
| β-actin | Bioss | BS-0061R | 1:5000(WB) | rabbit |
| pSTAT3 | Abways | CY6566 | 1:1000(WB) | rabbit |
| P-Akt | Abways | CY6569 | 1:1000(WB) | rabbit |
| AKT | Abways | CY5551 | 1:1000(WB) | rabbit |
| p-PI3K | Abways | CY6428 | 1:1000(WB) | rabbit |
| PI3 Kinase p110 | Abways | CY5161 | 1:1000/1:100 | rabbit |
| CD36 | Abways | CY5796 | 1:1000(WB) | rabbit |
| EGF | Abways | CY6818 | 1:1000(WB) | rabbit |
| STAT3 | Abways | CY5165 | 1:1000(WB) | rabbit |
| p-AMPK | Abways | CY6027 | 1:1000(WB) | rabbit |
| AMPK | Abways | CY5326 | 1:1000(WB) | rabbit |
| p-ERK1/2 | Abways | CY7165 | 1:1000(WB) | rabbit |
| ERK1/2 | Abways | CY5487 | 1:1000(WB) | mouse |
| ERα | Abways | CY8912 | 1:1000(WB) | rabbit |
| p-HSL | Abways | CY6274 | 1:1000(WB) | rabbit |
| Goat Anti-Rabbit | Abways | AB0151 | 1:3000 |  |
| Goat Anti-Mouse | Abways | AB0152 | 1:3000 |  |
