# Supplementary figures and images for: Melatonin improves endometrial receptivity and embryo implantation via MT2/PI3K/LIF signaling pathway in sows
Source: J Anim Sci Biotechnol. 2025 Jan 4;16:4. doi: 10.1186/s40104-024-01137-x (PMC11699789; doi:10.1186/s40104-024-01137-x)

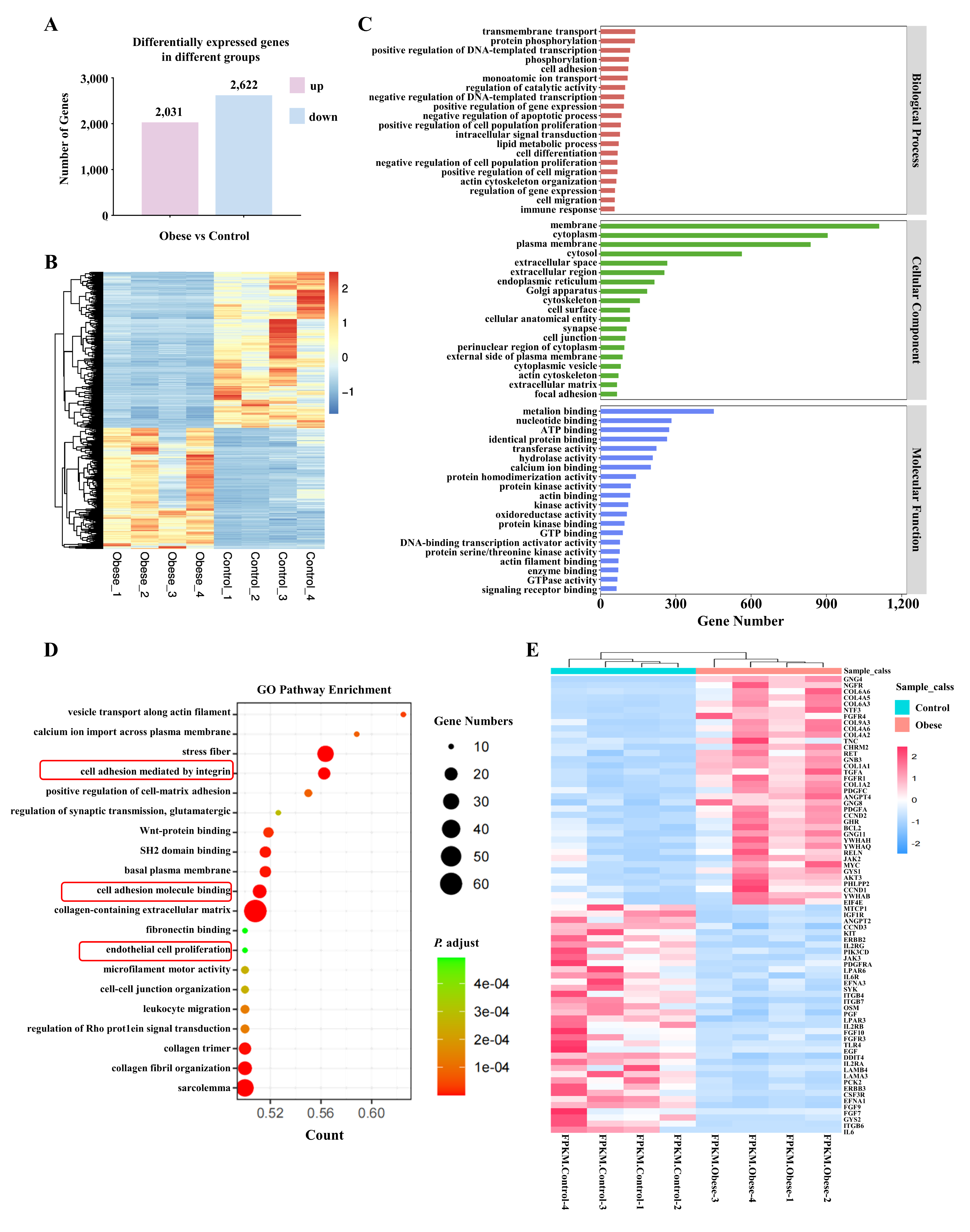

Supplement: Supplementary file 3 — Additional file 3: Fig. S1. Differential gene enrichment analysis with RNA-Seq. A Differentially expressed genes in two groups. B Uterine heat map. C–D GO enrichment. E Enrichment of the PI3K/AKT axis. **P< 0.01, q < 0.05. [file 40104_2024_1137_MOESM3_ESM.tif]

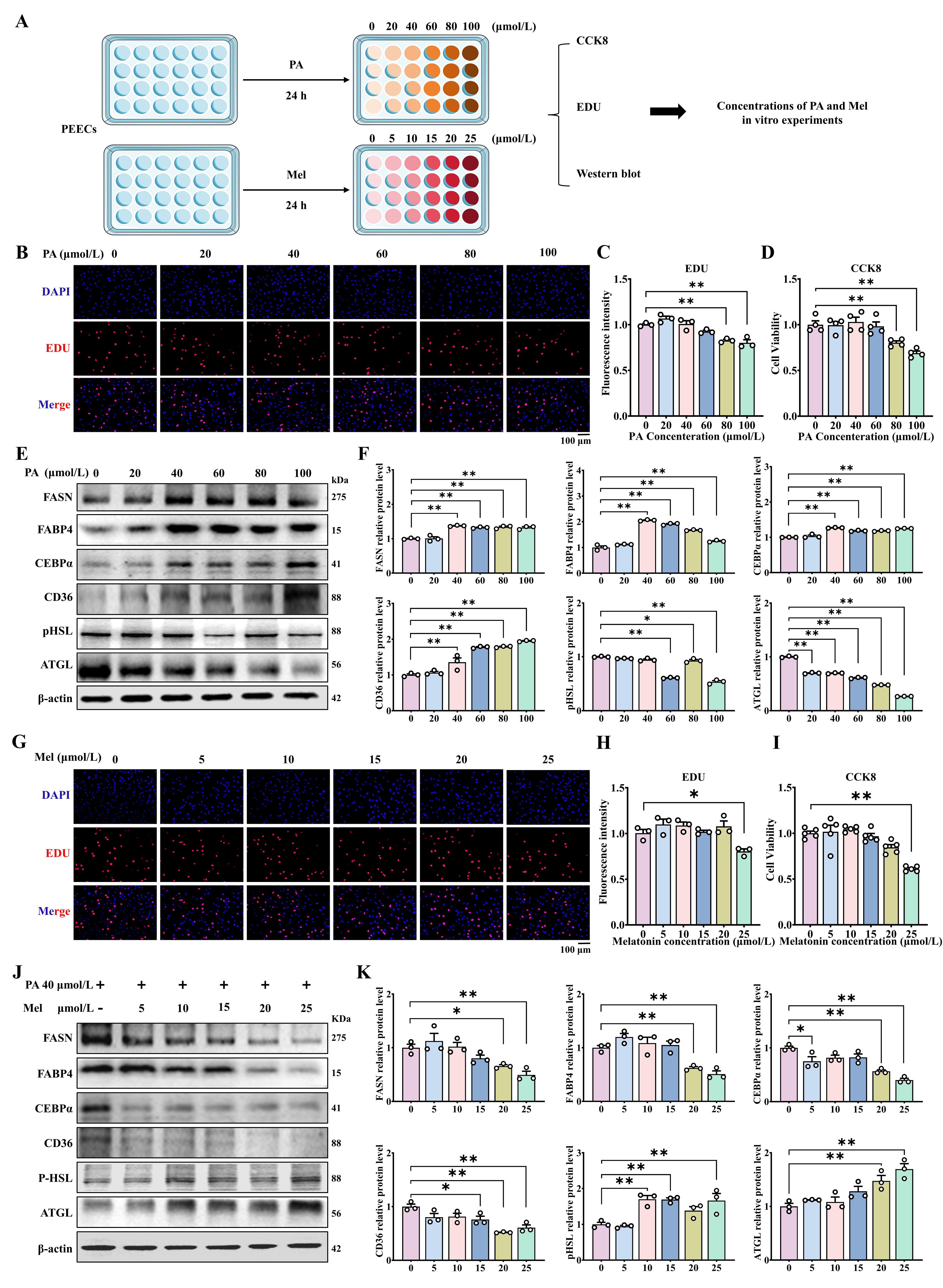

Supplement: Supplementary file 4 — Additional file 4: Fig. S2. Determination of PA and melatonin concentrations in vitro PEEC model. A Diagram showing the different concentrations of PA and melatonin used to treat PEECs. B Immunofluorescence staining with EdU (red) in PEECs (scale bar = 100 μm). C EdU-positive rate in PEECs (n = 3). D Cell viability detection (n = 4). E–F Western blotting evaluation of the relative protein expression of markers of lipogenesis (FASN, FABP4, CEBPα, and CD36) and lipolysis (pHSL and ATGL) (n = 3). G Immunofluorescence staining of EdU (red) in PEECs (scale bar = 100 μm). H EdU-positive staining rate (n = 3). Cell viability of PEECs (n = 4). J–K Western blotting analysis of the relative protein expression of markers of lipogenesis (FASN, FABP4, CEBPα, and CD36) and lipolysis (pHSL and ATGL) (n = 3). *P< 0.05; **P < 0.01. [file 40104_2024_1137_MOESM4_ESM.tif]

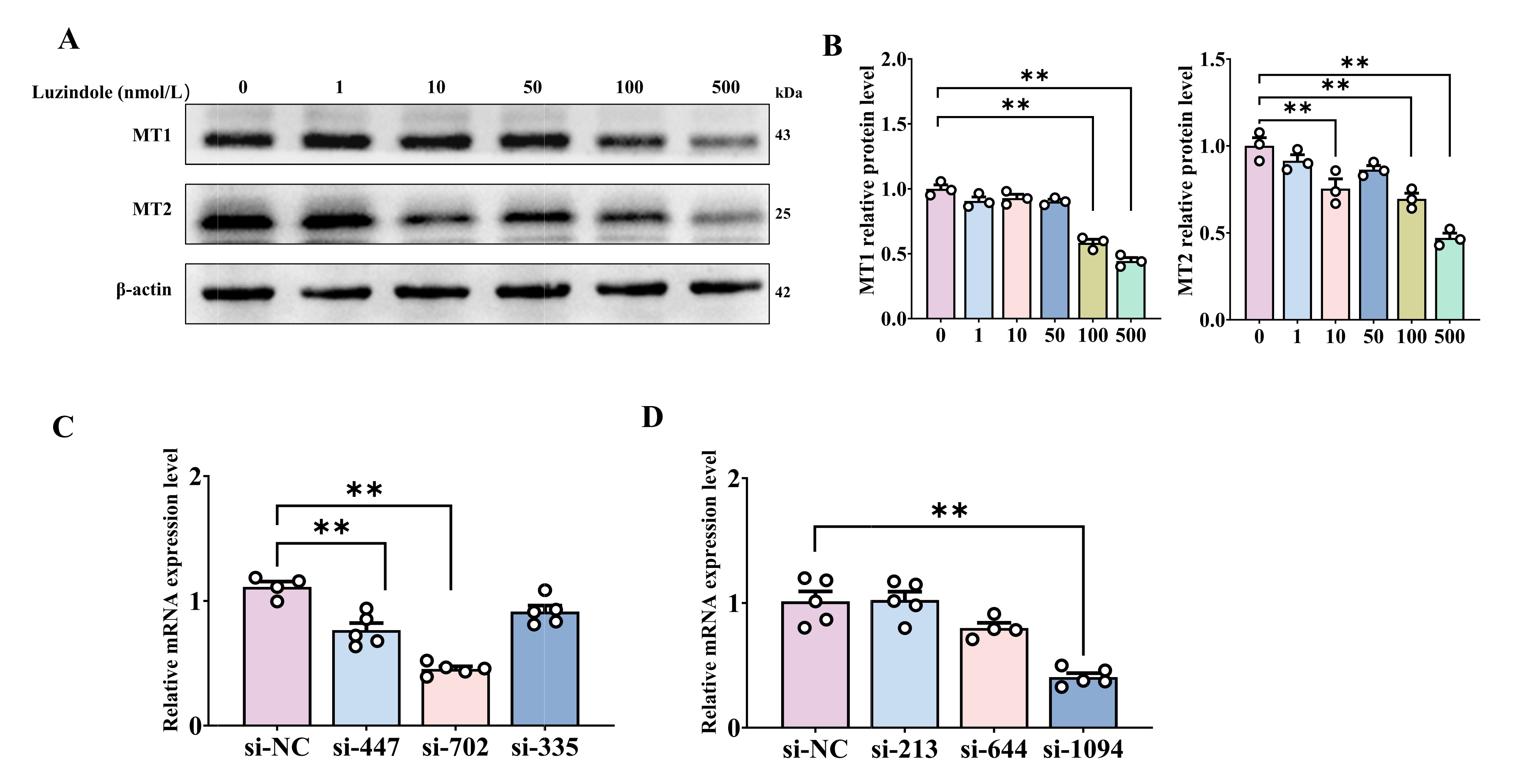

Supplement: Supplementary file 5 — Additional file 5: Fig. S3. Concentration screening of MT1 and MT2 inhibitor luzindole. A–B The relative protein expression of MT1 and MT2 (n = 3). C Quantification of MT1 interference efficiency (n = 5). D Quantification of MT2 interference efficiency (n = 5). *P < 0.05; **P< 0.01. [file 40104_2024_1137_MOESM5_ESM.tif]

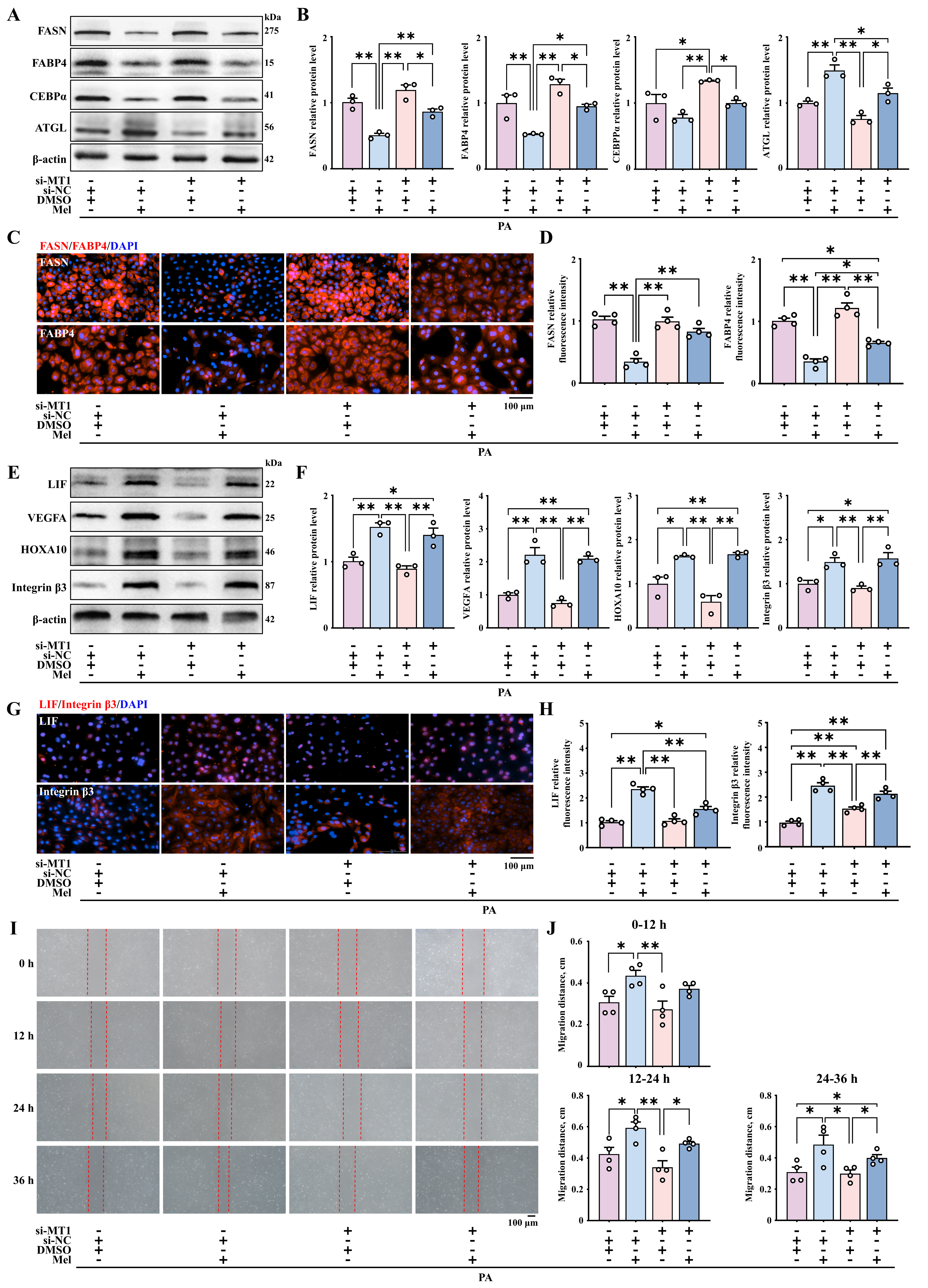

Supplement: Supplementary file 6 — Additional file 6: Fig. S4. No improvement in endometrial receptivity on melatonin binding to the MT1 receptor. A–B The relative protein expression of FASN, FABP4, and CEBPα and ATGL (n = 3). C Immunofluorescence staining of FASN and FABP4 (red) (scale bar = 100 μm). D Quantification of FASN and FABP4 expression (n = 4). E–F The relative protein expression of LIF, VEGFA, HOXA10 and Integrin β3 (n = 3). G Immunofluorescence staining of LIF and Integrin β3 (red) (scale bar = 100 μm). H Quantification of LIF and integrin β3 (n = 4). I Representative bright-field images showing the migration of PEECs at 0, 12, 24, and 36 h (scale bar = 100 μm). J Quantitative results of wound closure rates (n = 4). *P < 0.05; **P < 0.01. [file 40104_2024_1137_MOESM6_ESM.tif]

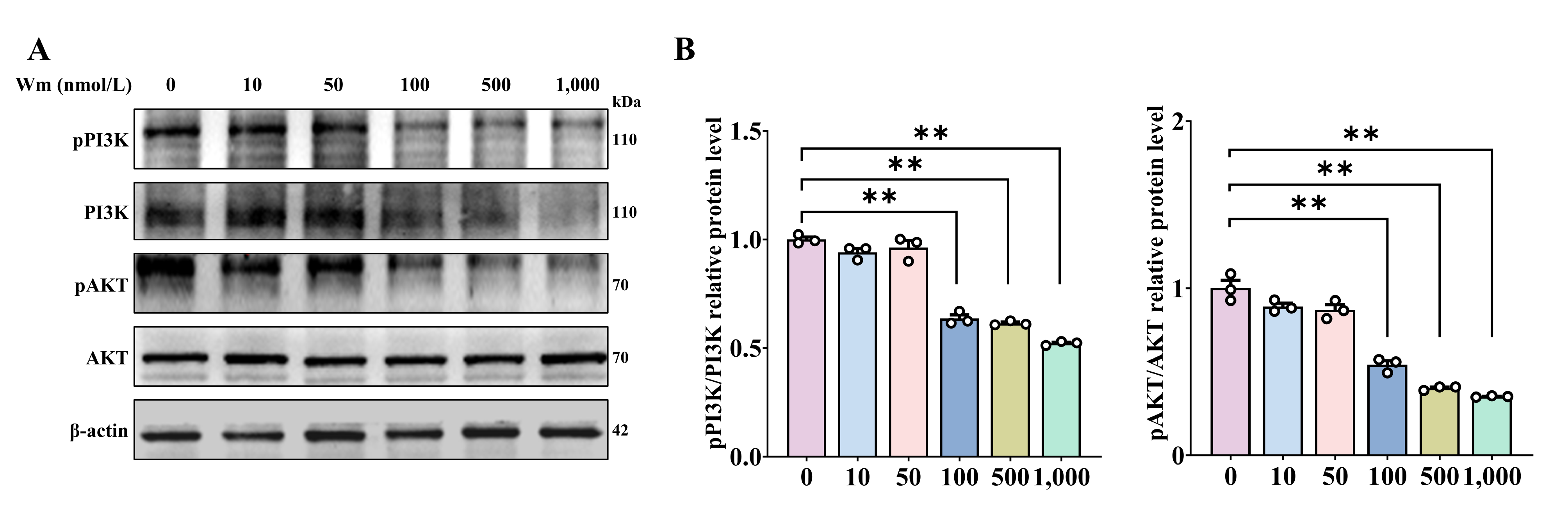

Supplement: Supplementary file 7 — Additional file 7: Fig. S5. Determination of the PI3K-specific inhibitor wortmannin. A–B The protein expression of pPI3K, PI3K, pAKT, and AKT in sows (n = 3). *P< 0.05; **P < 0.01. [file 40104_2024_1137_MOESM7_ESM.tif]

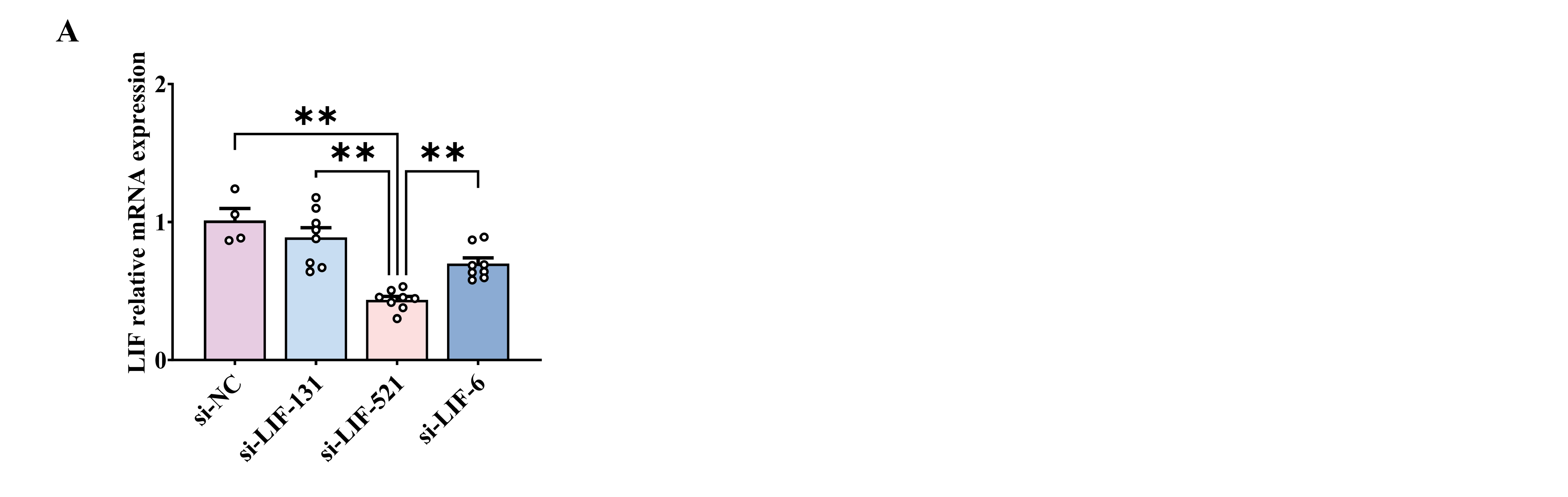

Supplement: Supplementary file 8 — Additional file 8: Fig. S6. Quantification of LIF interference efficiency (n = 8). *P < 0.05; **P < 0.01. [file 40104_2024_1137_MOESM8_ESM.tif]
